# The equine gastrointestinal microbiome: impacts of weight-loss

**DOI:** 10.1186/s12917-020-02295-6

**Published:** 2020-03-04

**Authors:** Philippa K. Morrison, Charles J. Newbold, Eleanor Jones, Hilary J. Worgan, Dai H. Grove-White, Alexandra H. Dugdale, Clare Barfoot, Patricia A. Harris, Caroline McGregor Argo

**Affiliations:** 1 Scotland’s Rural College, Craibstone Estate, Aberdeen, UK; 2 Scotland’s Rural College, Kings Buildings, Edinburgh, UK; 3 Aberystwyth University, Institute of Biological, Environmental and Rural Sciences, Aberystwyth University, Aberystwyth, UK; 4 University of Liverpool, Faculty of Health and Life Sciences, Leahurst Campus, Chester High Road, Neston, Wirral, UK; 5 ChesterGates Veterinary Specialists CVS (UK) Ltd., Chester, UK; 6 MARS Horsecare UK Ltd, Old Wolverton, Buckinghamshire, UK; 7 WALTHAM Petcare Science Institute, Freeby lane, Waltham-on-the-Wolds, Leicestershire, UK

**Keywords:** Equine, equine obesity, Weight-loss, Insulin dysregulation, Faecal microbiome, Apparent digestibility, Volatile fatty acid, Biomarkers

## Abstract

**Background:**

Obesity is an important equine welfare issue. Whilst dietary restriction is the most effective weight-loss tool, individual animals range in their weight-loss propensity. Gastrointestinal-derived bacteria play a fundamental role in host-health and have been associated with obesity and weight-loss in other species. This study evaluated the faecal microbiome (next-generation sequencing of 16S rRNA genes) of 15 obese Welsh Mountain pony mares, in the same 11-week period across 2 years (*n* = 8 Year 1; *n* = 7 Year 2). Following a 4-week acclimation period (pre-diet phase) during which time individuals were fed the same hay to maintenance (2% body mass (BM) as daily dry matter (DM) intake), animals underwent a 7-week period of dietary restriction (1% BM hay as daily DM intake). Faeces were sampled on the final 3 days of the pre-diet phase and the final 3 days of the dietary restriction phase. Bacterial communities were determined using Next Generation Sequencing of amplified V1-V2 hypervariable regions of bacterial 16S rRNA.

**Results:**

Losses in body mass ranged from 7.11 to 11.59%. Changes in the faecal microbiome composition following weight-loss included a reduction in the relative abundance of *Firmicutes* and *Tenericutes* and a reduction in indices of bacterial diversity. Pre-diet diversity was negatively associated with weight-loss. Pre-diet faecal acetate concentration was a strong predictor of subsequent weight-loss and negatively associated with *Sphaerochaeta (Spirochaetes* phylum) abundance. When animals were divided into 3 groups (high, mid, low) based overall weight loss, pre-diet bacterial community structure was found to have the greatest divergence between the high and low weight-loss groups (R = 0.67, *p* <  0.01), following PERMANOVA and ANOSIM analysis.

**Conclusions:**

Weight-loss in this group of ponies was associated with lower pre-diet faecal bacterial diversity and greater pre-diet acetate concentration. Overall, these data support a role for the faecal microbiome in weight-loss propensity in ponies and provide a baseline for research evaluating elements of the faecal microbiome in predicting weight-loss success in larger cohorts.

## Background

Bacteria residing in the gastrointestinal tract of all species play a fundamental role in host health and whole-body metabolism, whereby disruptions to energy balance, such as observed in obesity and associated metabolic diseases, are associated with dysbiosis of the gut microbiota [1–4]. Associations between obesity and the composition of the gut microbiome have been identified in humans [5, 6], dogs [7], and more recently our group have identified differences in faecal microbiome composition between lean, obese and aged ponies in the absence of differences in dietary provision [8].

Horses and ponies are adapted to consume high-fibre diets, whereby the microbial fermentation of dietary fibre in the hindgut produces volatile fatty acids (VFA) including acetate, propionate and butyrate that together contribute a significant proportion of the individual animals’ daily energy requirements [9]. A combination of increased nutrient-dense energy provisions and a lack of exercise has culminated in a high prevalence of obesity in leisure populations of horses and ponies in industrialised nations, and is a key welfare issue [10–12]. Specific breeds and types of horses and ponies (including UK native breeds such as Welsh breeds, Shetlands, Highlands etc.) have been identified to be more at risk of developing obesity than others [12], indicating a significant genetic component of obesity development in this species. However, as for humans, not all horses and ponies will lose weight at the same rate when placed on an energy restricted diet (1.25% body mass (BM) as dry matter (DM) daily), and some will require further restriction to 1.0% BM as DM daily to elicit expected reductions in BM [13].

Weight-loss achieved either through dietary restriction or by surgical methods in humans has been shown to induce significant alterations in the gut microbiome composition [14–18]. Obesity in man has previously been associated with a reduced proportion of the phylum *Bacteroidetes* and correspondingly increased proportion of *Firmicutes*, which can be reversed following a period of diet-induced weight-loss [6]. However, this appears to be an inconsistent finding between studies [15, 19, 20], and findings from a recent rodent meta-analysis has suggested that the *Bacteroidetes*-to-*Firmicutes* ratio may not be a useful marker of obesity [21]*.* More recently, in humans, a higher pre-treatment ratio of *Prevotella*-to-*Bacteroides* was found to result in greater losses of body weight and body fat on a high-fibre diet [22]. Furthermore, although specific bacterial groups have been associated with the degree of weight-loss achieved during dietary-restriction in human subjects, methodological differences may partly explain inconsistent findings between studies [15, 17].

Obesity is often accompanied by metabolic disorders including insulin dysregulation, which itself has been associated with altered microbiome composition and reduced bacterial richness in humans [23]. A greater outset abundance of *Akkermansia muciniphila* was found to be associated with greater improvements in markers of insulin sensitivity, total and LDL cholesterol, and greater reductions in waist-to-hip ratio in humans following weight loss through dietary restriction [24]. Recently, evidence has been provided to indicate that differences in the gut microbiome composition among genetically different strains of mice confers differing capacities to digest dietary sugars, in turn altering the susceptibility of different mouse strains to metabolic disease through links between microbial metabolism and insulin secretion [25].

To date, studies evaluating the gut microbiome in the horse have generally focused on the impact of diet [26–28]. Additionally, associations between the faecal microbiome and chronic laminitis [29] and equine metabolic syndrome (EMS) [30] have been evaluated. Although no studies have yet been conducted to describe changes in the composition of the faecal microbiome following weight-loss in ponies, two studies have evaluated the stability of the faecal microbiome in groups of horses and ponies across time. Across a 6-week period at the end of a weight-loss trial, it was identified that 65% of the faecal bacterial community in a group of horses was retained between time points [31], and more recently, the stability of the equine faecal microbiome over a 52-week period was evaluated in a small population of horses maintained at pasture [32]. Therefore, we have some evidence that as for other species the equine gut microbiome is influenced by diet and by different disease states. The objective of the current study was to evaluate the change in faecal microbiome composition following dietary restriction in obese ponies and to identify any associations between microbiome composition and weight-loss propensity. To minimise the effect of potential confounding factors, the animals used in the current study were all the same breed (Welsh Section A) and sex (mare).

## Results

### Changes in body mass and body composition

All animals remained healthy throughout the study. One animal lost a considerably greater amount of BM compared to the other animals (3.68% of outset BM) during the ‘pre-diet’ phase of the study (Week − 4 to 0). During this time, individual animals were fed 2% BM as hay DM daily, designed to approximate maintenance levels. Therefore, due to suspected underlying dental issues, data from this animal were removed from all analysis. The remaining 15 animals generally held a constant BM during the ‘pre-diet’ phase of the study (overall mean gain of 0.11% ± 1.93).

The rate of weight-loss during the dietary restriction phase of the study (Week 0 to 7; animals fed 1% BM as hay DM daily) was greatest during the first week (overall mean loss 2.82% ± 1.64). During the remaining 6 weeks, animals lost an average of 1.01% ± 0.21 Week 1 BM weekly, leading to overall losses of 6.04% ± 1.23 over the 6 weeks.

**Fig. 1:**
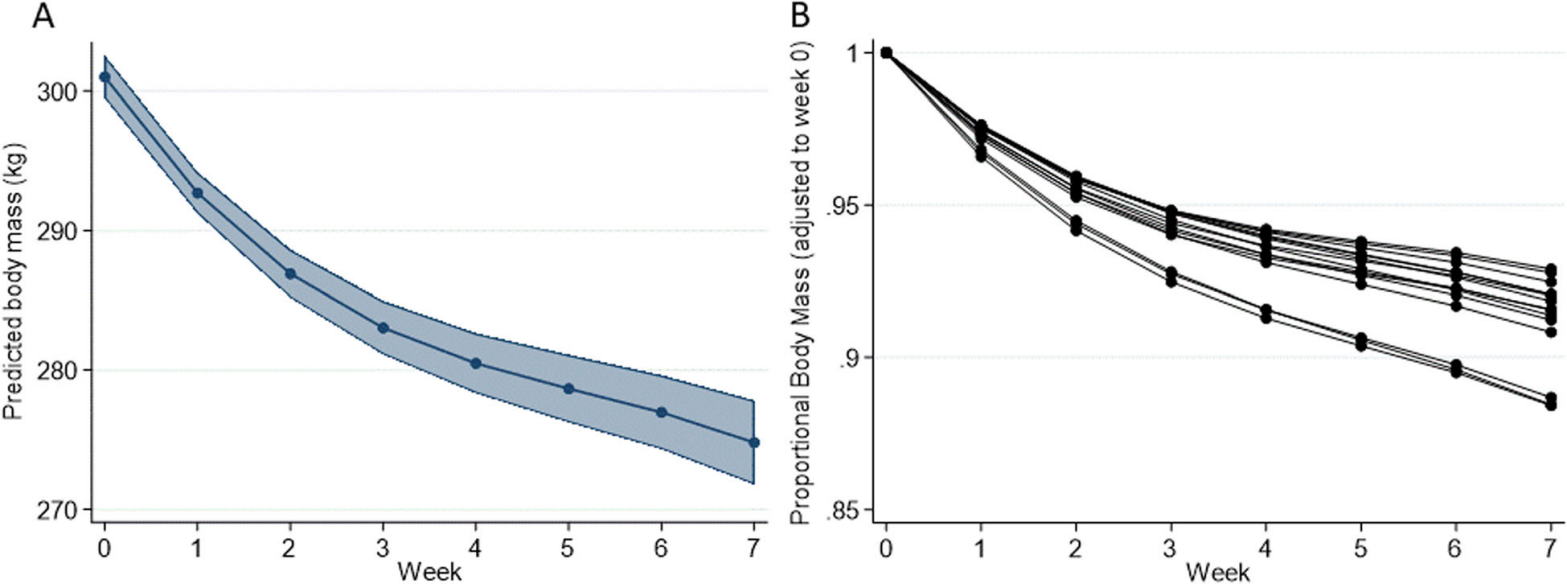
Changes in body mass over the 7-week dietary restriction phase of the study. (A) Marginal mean plot derived predicted body mass change (*n* = 15). Shaded area corresponds to 95% confidence intervals. (B) Predicted cumulative proportional weight-loss for individual animals (adjusted to Week 0; n = 15)

The final regression model (Table 1) for investigating changes in body mass over the weight-loss phase of the study included week as a cubed polynomial term. Predicted marginal mean bodyweight was estimated from the regression model and is presented graphically (Fig. 1a) and the predicted cumulative proportional weight-loss (adjusted to Week 0) were plotted for individual animals (Fig. 1b). Using the predicted model outputs, animals were ranked according to the degree of weight-loss achieved (Table 2).

**Table 1:** Change in body mass of obese ponies during 7-weeks of dietary restriction

Explanatory variable	Coefficient	95% CI	*P* value
Week	−9.78	−11.31 to −8.25	< 0.01
Week^2^	1.56	1.03 to 2.09	< 0.01
Week^3^	−1.00	−0.15 to − 0.05	< 0.01
Start weight	1.00	0.96 to 1.03	< 0.01
Baseline	0.69	−10.36 to 11.75	0.90
Random effects parameter			
Pony ID: Unstructured			
Variance (week)	0.25	0.09 to 0.70	
Variance (baseline)	4.32	1.47 to 12.67	
Covariance (week, baseline)	1.03	0.24 to 1.82	
Variance (residual)	5.63	4.29 to 7.39

**Table 2:** Outset animal phenotype including ranking of animals by weight-loss (WL)

Ranking	Pony ID	Year	% WL	Low/mid/high WL	Age (years)	BM (kg)	BCS (/9)	Body fat (%)
1	10	2	**11.59**	high	9	244	7.7	17.19
2	16	2	**11.56**	high	7	282	7.6	12.30
3	11	2	**11.32**	high	6	260	7.6	14.60
4	14	2	**9.17**	high	10	310	8.2	21.59
5	15	2	**8.77**	high	5	315	8.1	20.54
6	12	2	**8.62**	mid	12	351	8.2	24.64
7	8	1	**8.45**	mid	23	279	7.1	20.02
8	7	1	**8.42**	mid	20	261	7.3	22.03
9	13	2	**8.15**	mid	13	353	8.4	20.67
10	2	1	**7.97**	mid	11	283	8.1	27.32
11	4	1	**7.94**	low	10	335	7.8	16.95
12	5	1	**7.94**	low	11	347	8.3	21.65
13	1	1	**7.53**	low	10	318	8	24.61
14	3	1	**7.22**	low	11	291	8.2	23.33
15	6	1	**7.11**	low	8	289	7.3	27.78

A mixed-effects linear regression model was built (random effects: Pony ID, random slope: week) with body mass (BM) as the outcome variable and week as a cubic polynomial as the explanatory variable. An unstructured covariance matrix was employed for the random effects. Coefficients are presented ±95% confidence intervals (CI’s).

Body mass was predicted from the mixed-effects model and using this, a cumulative proportional weight loss (adjusted to Week 0) was calculated and used to rank animals according to weight-loss.

The proportion of BM occupied by fat mass remained similar before and after dietary-restriction (21.01% ± 4.38 vs. 21.22% ± 4.40; Table 3), however due to reductions in overall BM, fat mass was correspondingly significantly reduced (63.75 kg ± 16.13 vs. 58.92 kg ± 14.74; *p* < 0.01). When losses of lean and fat mass were evaluated as a proportion of outset lean/fat masses, reductions in these compartments were similar (7.32% ± 4.74 fat mass loss vs. 8.70% ± 2.32 lean mass loss; Table 3). There were no associations between outset body fat percentage and percentage of weight lost as fat mass or lean mass.

**Table 3:** Summary data for pre- and post-diet combined glucose/insulin tests (CGIT), digestibility trials and body composition data

	Outset (pre-diet) Mean ± SD	End (post-diet) Mean ± SD
Insulin/glucose dynamics		
Baseline insulin (mU/L)	10.35 ± 6.77	7.00 ± 3.27*
Insulin 45 min post-infusion (mU/L)	217.41 ± 104.69	256.34 ± 97.77
Insulin 75 min post-infusion (mU/L)	105.62 ± 124.06	143.81 ± 77.15
Area under curve insulin (mU/L/min)	9970.23 ± 5653.16	11,927.61 ± 4785.72
Area under curve glucose (mmol/L/min)	896.27 ± 138.90	1040.49 ± 99.89*
Return to baseline glucose (minutes)	73.00 ± 40.30	123.33 ± 27.69*
Digestibility		
Gross energy digestibility (%)	48.27 ± 5.76	49.67 ± 3.11
Dry matter digestibility (%)	50.20 ± 4.90	51.90 ± 3.39
Neutral detergent fibre digestibility (%)	49.37 ± 7.36	49.31 ± 5.35
Body composition		
Body mass (kg)	301.20 ± 34.83	275.20 ± 33.59*
Lean mass (kg)	237.71 ± 27.13	217.15 ± 26.36*
Fat mass (kg)	63.75 ± 16.13	58.92 ± 14.74*
Body fat (%)	21.01 ± 4.38	21.22 ± 4.40
Fat loss as % of outset fat mass (%)		7.32 ± 4.74
Lean loss as % of outset lean mass (%)		8.70 ± 2.32

Body composition was calculated from deuterium oxide dilution tests. *indicates significant differences (p < 0.05) from pre-diet values. SD, standard deviation.

### Digestibility and glucose/insulin dynamics

Mean apparent digestibilities of GE, DM, and NDF were not altered following the dietary restriction (Table 3). Positive associations were found between pre-diet digestibilities (GE, DM, NDF) and subsequent weight-loss (Additional file 1). Additionally, positive associations were identified between the change in NDF digestibility (pre-diet minus post-diet) and overall weight-loss (Additional file 1), whereby animals whose apparent NDF digestibility reduced following weight-loss had greater overall weight-loss than those animals whose apparent NDF digestibility was increased following weight-loss. Glucose:insulin dynamics as measured by the CGIT revealed some differences following weight loss. Baseline plasma insulin values were significantly reduced following dietary restriction, but all remained within acceptable normoinsulinaemic ranges (*p* < 0.05; 10.35μIU/ml ± 6.77 pre-diet to 7.00 μIU/ml ± 3.27 post-diet; Table 3). Insulin measured at time 45 and 75 min, and by default, area under the curve for insulin from the CGIT tended to increase following dietary restriction, but did not reach statistical significance (Table 3). Baseline glucose values were not significantly changed following weight-loss, however the area under the curve for glucose response from the CGIT was significantly increased (*p* < 0.01; 896.27 mmol/L/min ± 138.90 pre-diet to 1040.49 mmol/L/min ± 99.89 post-diet; Table 3), as was the time to return to baseline for glucose (p < 0.01; 73.00 min ± 40.30 pre-diet to 123.33 min ± 27.69 post-diet; Table 3).

### Coverage, diversity and volatile fatty acids

Quality filtering of 16S rDNA amplicon sequences resulted in 16,028,420 high-quality sequences (320 bp long) which clustered in 9536 different OTUs. A phylogenetic tree was constructed (PRIMER 6 with Bray Curtis dissimilarity, Additional file 2), which indicated that samples from an animal, collected on each of the three successive sampling days pre- and post-diet, tended to cluster together. This observation allowed data arising from these serial samples to be pooled for each animal. Pooled analyses provided 14,500 sequences per animal, per period after normalization. Rarefaction curves (Additional file 3) demonstrated that sample curves had not plateaued; indicating that complete sampling of these environments had not yet been achieved.

**Fig. 2:**
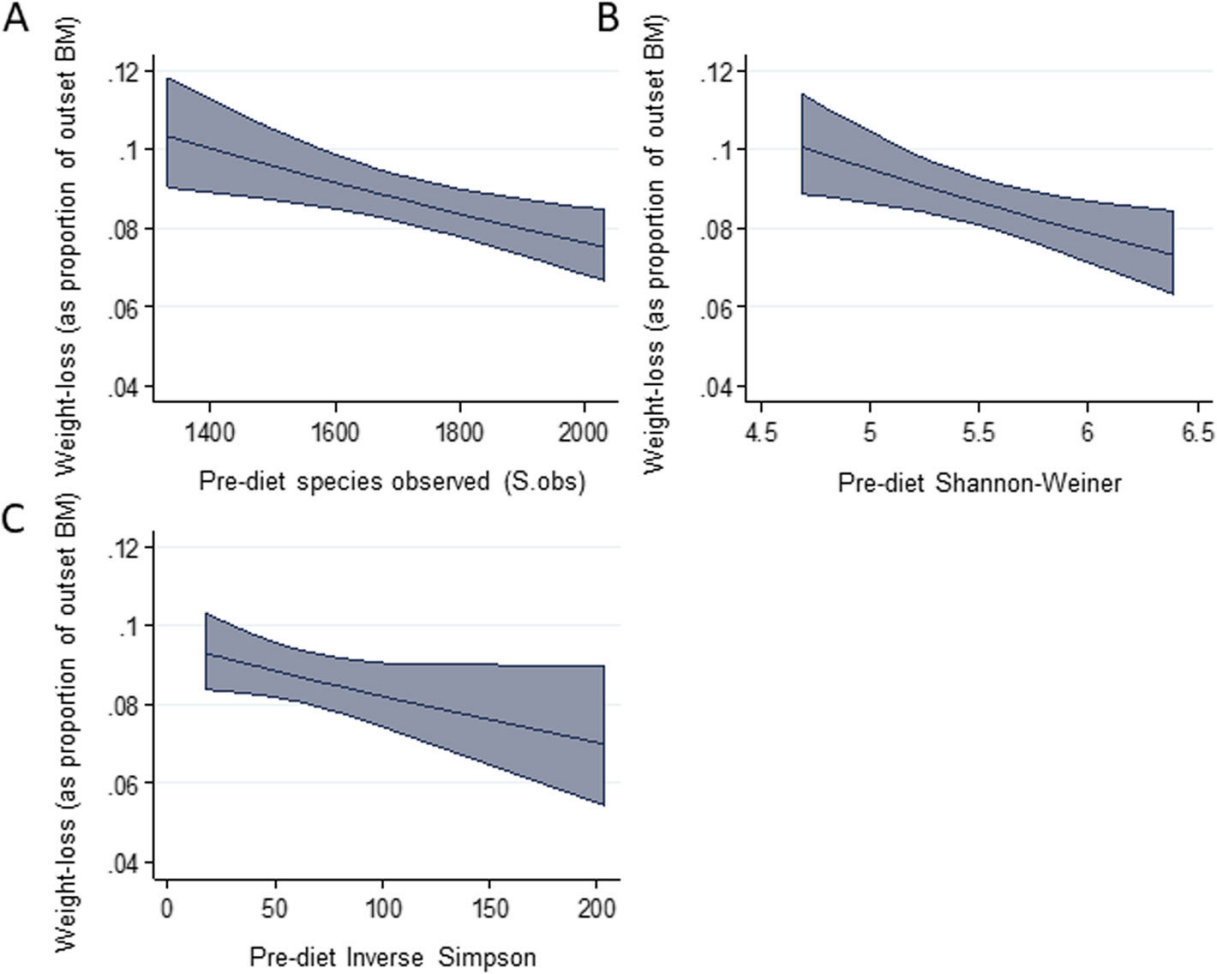
Marginal mean plots illustrating associations between overall predicted proportional weight-loss and pre-diet diversity indices: (A) Species observed, (B) Shannon-Weiner, (C) Inverse Simpson. Shaded area corresponds to 95% CI. n = 15

There were significant (*p* < 0.05) reductions in Inverse Simpson, Shannon-Wiener, and S.obs measures of alpha diversity following weight-loss, but no differences were observed for Chao1 (Table 4). Furthermore, there were significant differences in pre-diet (mean of 3 pre-diet days) measures of diversity (Inverse Simpson, Shannon-Wiener, and S.obs) between the three weight-loss groups (Table 4). This was confirmed by univariate regression analysis, whereby significant negative associations were identified between pre-diet diversity measures and subsequent weight-loss (Additional file 4 and Fig. 2).

**Table 4:** Effect of weight-loss on bacterial diversity and difference in pre-diet diversity between weight-loss groups

	Pre-diet	Post-diet	*P*-value	High	Medium	Low	*P*-value
Inverse Simpson	62.02 ± 47.66	27.03 ± 16.91	< 0.01	28.31 ± 13.14	54.10 ± 21.77	103.65 ± 60.32	0.03
Shannon-Weiner	5.46 ± 0.52	4.92 ± 0.54	< 0.01	4.94 ± 0.28	5.55 ± 0.35	5.95 ± 0.29	< 0.01
S.Obs	1717.33 ± 222.51	1576.20 ± 153.13	0.04	1488.93 ± 106.99	1748.27 ± 156.16	1914.80 ± 149.37	< 0.01
S.Chao1	2871.63 ± 401.75	2729.61 ± 324.73	0.37	2619.25 ± 279.27	2986.81 ± 552.08	3008.82 ± 249.78	0.24

Data presented are mean ± SD. Diversity indices were calculated at both pre- and post-diet time-points (*n* = 15), and pre-diet diversity indices (mean of 3 pre-diet days) are presented for the high (*n* = 5), medium (n = 5) and low (n = 5) weight-loss groups.

**Fig. 3:**
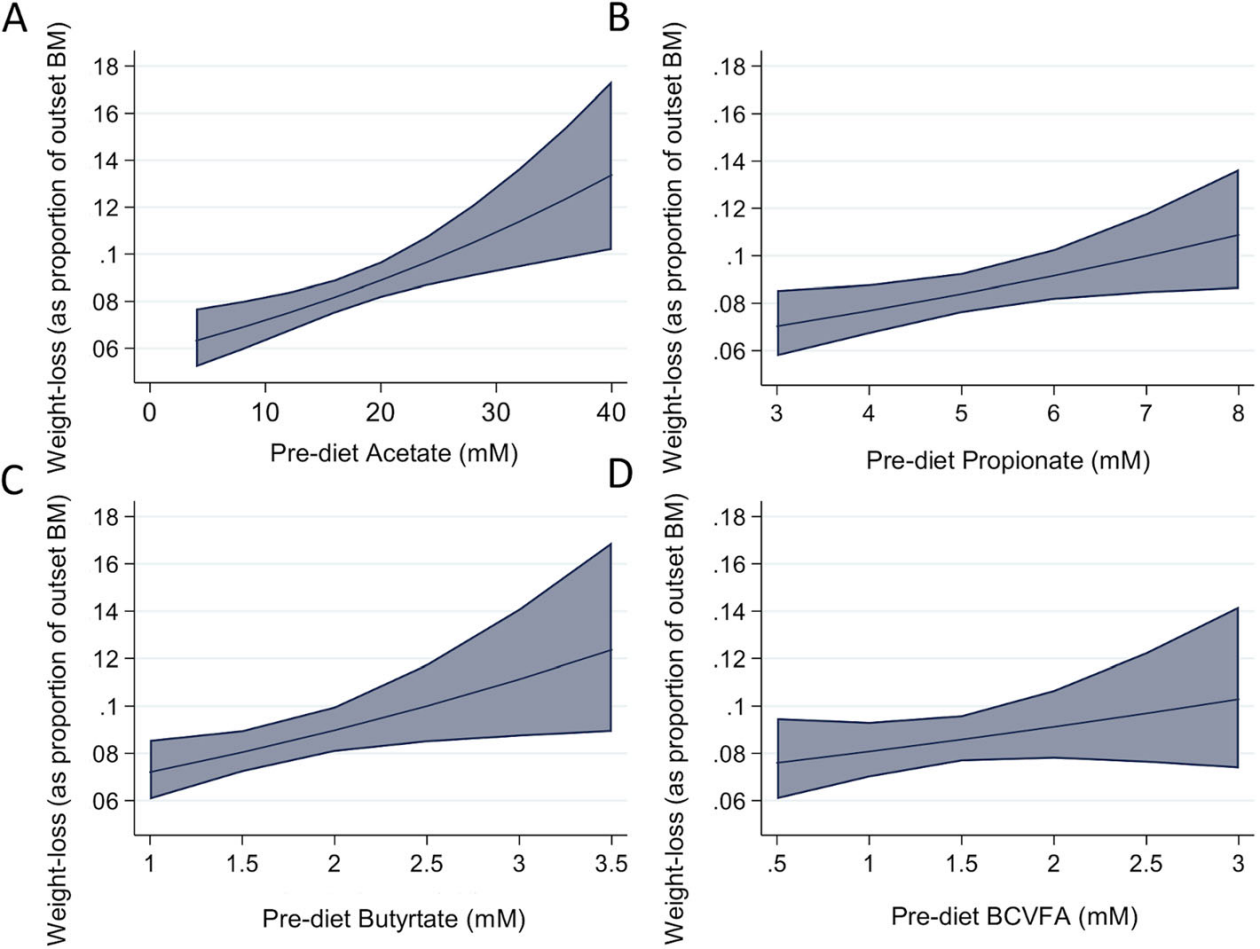
Marginal mean plots illustrating pre-diet VFA concentrations: (A) Acetate, (B) Propionate, (C) Butyrate, (D) Branched chained volatile fatty acids (BCVFA). Shaded area corresponds to 95% CI. n = 15

There were significant reductions in the concentrations of acetate, butyrate, propionate and branched chain volatile fatty acids (BCVFA; comprised of isobutyrate, isovalerate, isocaproic acid and heptanoic acid) following weight-loss, however faecal pH remained unchanged (Table 5). There were no differences in the ratio of acetate plus butyrate to propionate between the pre-diet and post-diet samples, however the pre-diet ratio (mean of 3 pre-diet samples) was greatest in the high weight-loss group compared to the low weight-loss group (4.36 ± 0.58 vs. 3.24 ± 0.50; *p* = 0.04). Additionally, strong positive associations were identified between pre-diet VFA concentrations and subsequent weight-loss (Acetate, R^2^ = 0.51, *p* < 0.01; Butyrate, R^2^ = 0.28, *p* = 0.02; Propionate, R^2^ = 0.27, *p* = 0.03; Additional file 5 and Fig. 3).

**Table 5:** Effect of weight-loss on faecal dry matter content, pH and volatile fatty acid concentrations and differences in pre-diet faecal dry matter content, pH and VFA concentrations between weight-loss groups

	Pre-diet	Post-diet	*P*-value	High	Medium	Low	*P*-value
Faecal dry matter (%)	16.78 ± 1.44	18.98 ± 1.96	< 0.01	16.74 ± 1.38	16.53 ± 1.14	17.07 ± 1.97	0.85
pH	6.73 ± 0.24	6.72 ± 0.15	0.90	6.74 ± 0.04	6.77 ± 0.10	6.69 ± 0.43	0.90
Acetate (mM)	18.04 ± 7.06	14.39 ± 8.35	< 0.01	24.04 ± 6.76	17.37 ± 6.58	12.70 ± 2.04	0.02
Propionate (mM)	5.19 ± 1.33	3.66 ± 1.71	< 0.01	5.98 ± 1.31	5.16 ± 1.56	4.42 ± 0.74	0.19
Butyrate (mM)	1.77 ± 0.55	1.45 ± 0.88	0.04	2.09 ± 0.73	1.73 ± 0.45	1.48 ± 0.29	0.22
BCVFA (mM)	1.44 ± 0.58	1.13 ± 0.85	0.05	1.56 ± 0.88	1.55 ± 0.51	1.22 ± 0.18	0.59

Data presented are mean ± SD. Faecal pH and VFA concentrations were measured at both pre- and post-diet time-points (*n* = 15), and pre-diet values (mean of 3 pre-diet days) in the high (*n* = 5), medium (n = 5) and low (n = 5) weight-loss groups. BCVFA: branched-chain volatile fatty acids.

Due to the significant association between pre-diet acetate and subsequent weight-loss, a logistic regression model was fitted with the binary outcome of “weight loss ≥ 8% BM”. This was used to construct a receiver operating characteristic (ROC) curve to determine the ability of pre-diet acetate concentration to predict subsequent weight-loss success (using target weight-loss of 8% pre-diet body mass) and resulted in an area under the curve of 0.89 (Additional File 6). Optimal test performance achieved with the current data provided a cut-off value of 14 mM acetate which gave a test sensitivity of 88.9% (95% confidence intervals: 77.4–95.8) and specificity of 88.3% (95% confidence intervals: 67.2–93.6) for prediction of weight loss greater than 8%. The pre-diet abundance of *Sphaerochaeta, Treponema,* (both belonging to the *Spirochaetes* phylum), *Anaerovorax* and *Mobilitalea* (both belonging to *Firmicutes* phylum) were found to be significantly different between pre-diet acetate concentrations (divided into quartiles; Additional File 7) whereby the pre-diet abundance of the individual genera was greatest for those animals in the lowest quartile of pre-diet acetate concentration.

### Changes in bacteria abundance following weight-loss and associations with weight-loss

**Fig. 4:**
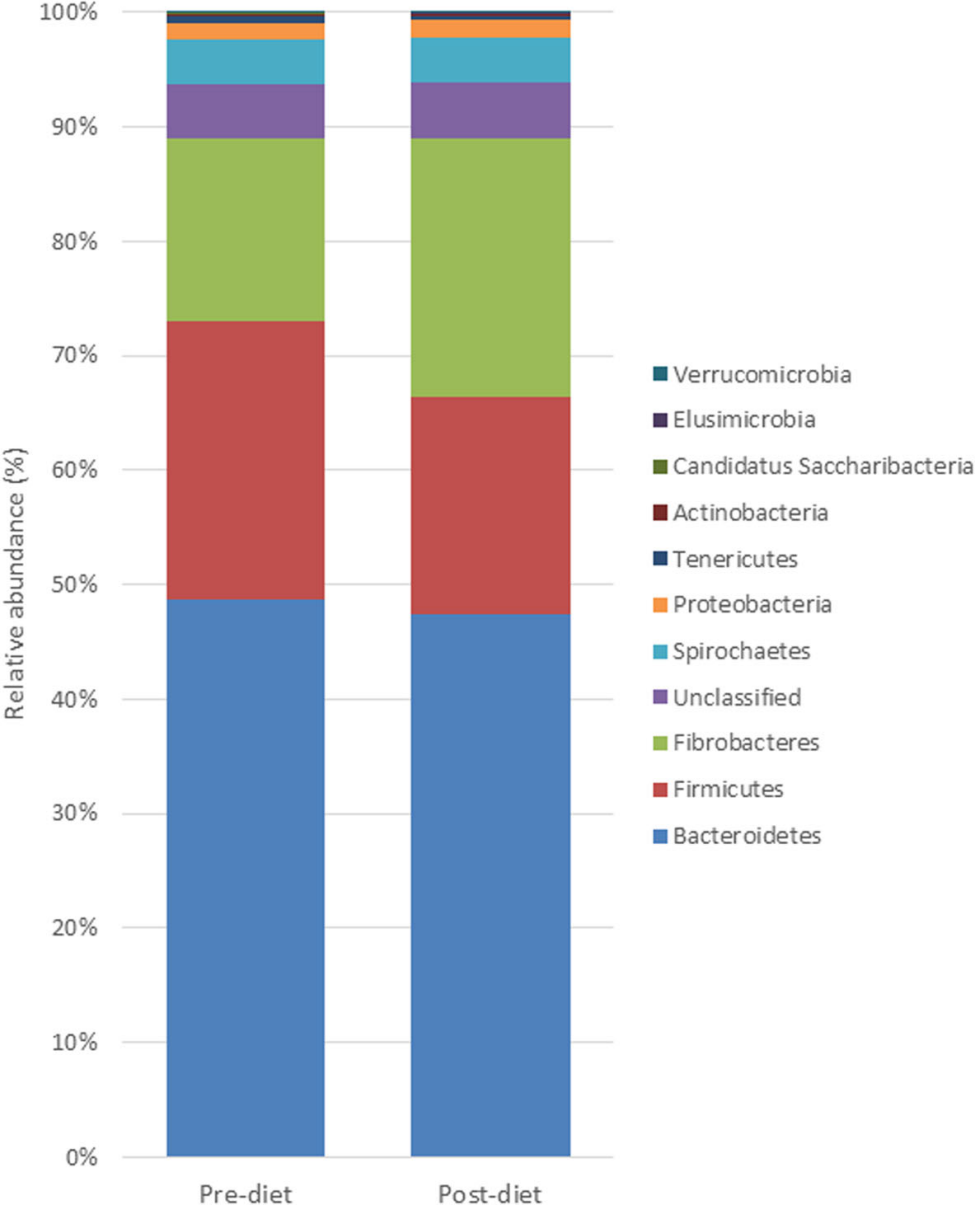
Stacked histogram illustrating the relative abundance of the dominant bacterial phyla (present at > 0.05% relative abundance) before (pre-diet), and after 7 weeks of dietary restriction (post-diet; n = 15)

Across all samples/time-points, *Bacteroidetes* were the most abundant phylum present, followed by the *Firmicutes* and *Fibrobacteres.* The relative abundance of *Firmicutes, Tenericutes* and *Elusimicrobia* were significantly reduced following weight-loss (Table 6 and Fig. 4), and although the relative abundance of *Bacteroidetes* did not change significantly following weight-loss, the *Bacteroidetes:Firmicutes* ratio was increased following weight-loss (2.10 ± 0.54 pre-diet to 2.54 ± 0.56 post-diet; *p* = 0.01). Similarly, the ratio of the fibre-digesting bacteria, *Fibrobacteres:Firmicutes* was increased following weight-loss (0.74 ± 0.58 to 1.31 ± 0.88; *p* = 0.03). The pre-diet abundance of *Actinobacteria* and *Spirochaetes* were found to be significantly greater in the low weight-loss group compared to the high weight-loss group (Additional File 8).

**Table 6:** Relative abundance of bacterial phyla before (pre-diet), and after 7 weeks of dietary restriction (post-diet; *n* = 15)

	Pre-diet	Post-diet	SED	Benjamini-Hochberg *P*-value
*Firmicutes*	0.2420	0.1894	0.015	0.007
*Tenericutes*	0.0062	0.0029	0.006	0.007
*Elusimicrobia*	0.0006	0.0014	0.003	0.007
*Verrucomicrobia*	0.0003	0.0008	0.005	0.121
*Fibrobacteres*	0.1602	0.2264	0.041	0.187
*Candidatus Saccharibacteria*	0.0008	0.0006	0.002	0.198
*Bacteroidetes*	0.4874	0.4740	0.017	0.697
*Unclassified*	0.0468	0.0487	0.008	0.697
*Proteobacteria*	0.0149	0.0158	0.008	0.754
*Spirochaetes*	0.0389	0.0383	0.01	0.997
*Actinobacteria*	0.0018	0.0018	0.002	0.997

**Fig. 5:**
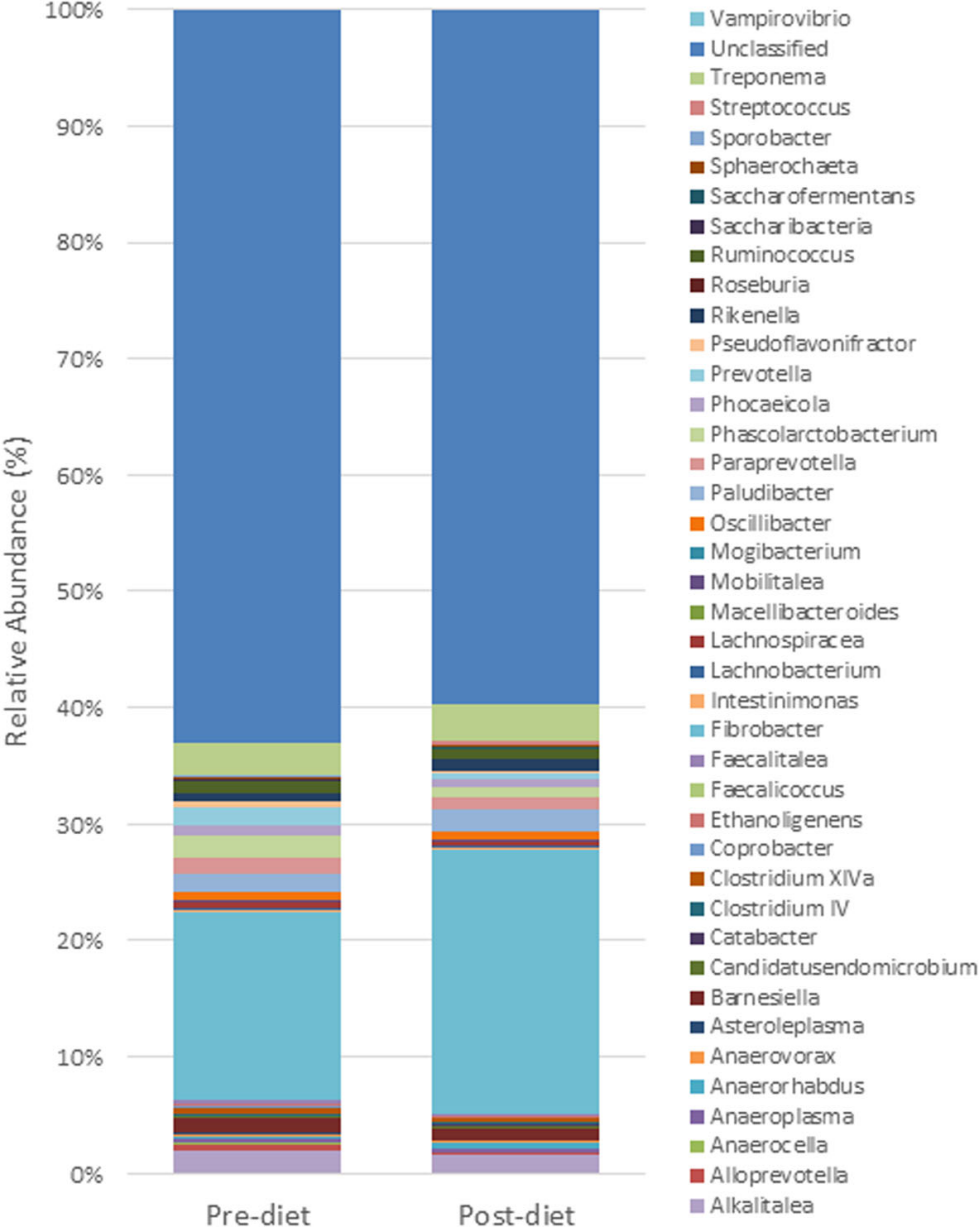
Stacked histogram illustrating the relative abundance of the dominant bacterial genera (present at > 0.05% relative abundance) before (pre-diet), and after 7 weeks of dietary restriction (post-diet; n = 15)

At the genera level, whilst the largest proportions of samples were unclassified at this level (belonging to 33 different families, Additional File 9), *Fibrobacter* was the second-most abundant genus across all samples/time-points. A total of 19 individual genera were altered in abundance following weight-loss: 12 were reduced in abundance, whilst the remaining 7 increased in abundance following weight-loss (Table 7 and Fig. 5). The pre-diet abundance of *Mobilitalea* genus (belonging to the *Firmicutes* phylum) was found to be significantly greater in low compared to high weight-loss groups (Additional File 10).

**Table 7:** Relative abundance of bacterial genera before (pre-diet), and after 7 weeks of dietary restriction (post-diet; *n* = 15)

	Pre-diet	Post-diet	SED	Benjamini-Hochberg *P*-value
*Phascolarctobacterium*	0.019	0.009	0.009	0.005
*Prevotella*	0.016	0.007	0.008	0.005
*Anaeroplasma*	0.004	0.002	0.004	0.005
*Coprobacter*	0.002	0.001	0.005	0.005
*Roseburia*	0.001	0.000	0.002	0.005
*Anaerorhabdus*	0.001	0.006	0.008	0.005
*Candidatusendomicrobium*	0.000	0.001	0.003	0.005
*Catabacter*	0.000	0.001	0.003	0.005
*Clostridium XIVa*	0.006	0.004	0.004	0.009
*Alloprevotella*	0.005	0.001	0.007	0.012
*Streptococcus*	0.001	0.002	0.005	0.022
*Rikenella*	0.007	0.011	0.006	0.024
*Pseudoflavonifractor*	0.005	0.000	0.012	0.035
*Saccharofermentans*	0.001	0.001	0.003	0.035
*Faecalitalea*	0.002	0.001	0.006	0.066
*Anaerovorax*	0.002	0.001	0.003	0.090
*Lachnospiracea*	0.005	0.004	0.005	0.099
*Paraprevotella*	0.013	0.010	0.008	0.103
*Faecalicoccus*	0.001	0.000	0.003	0.111
*Anaerocella*	0.001	0.001	0.003	0.111
*Macellibacteroides*	0.001	0.000	0.005	0.125
*Lachnobacterium*	0.002	0.001	0.003	0.134
*Fibrobacter*	0.161	0.228	0.042	0.150
*Ruminococcus*	0.010	0.008	0.009	0.172
*Asteroleplasma*	0.002	0.001	0.006	0.172
*Saccharibacteria*	0.001	0.001	0.002	0.172
*Mogibacterium*	0.001	0.001	0.001	0.175
*Phocaeicola*	0.009	0.006	0.009	0.185
*Sphaerochaeta*	0.001	0.002	0.003	0.185
*Rreponema*	0.028	0.031	0.008	0.216
*Alkalitalea*	0.020	0.016	0.028	0.287
*Unclassified*	0.629	0.597	0.017	0.300
*Sporobacter*	0.002	0.002	0.002	0.354
*Mobilitalea*	0.001	0.001	0.003	0.740
*Paludibacter*	0.016	0.019	0.019	0.994
*Barnesiella*	0.014	0.011	0.018	0.994
*Osscillibacter*	0.007	0.007	0.004	0.994
*Intestinimonas*	0.002	0.002	0.005	0.994
*Clostridium IV*	0.002	0.002	0.004	0.994
*Ethanoligenens*	0.001	0.001	0.006	0.994
*Vampirovibrio*	0.001	0.001	0.003	0.994

A total of 61 OTUs belonging to 13 genera were found to be significantly different in abundance following weight-loss: 7 of which were decreased in abundance following weight-loss and the remaining 51 were increased in abundance (Additional File 11). Significant differences in pre-diet OTU abundances were also identified between the weight-loss groups: the greatest numbers of differences were identified between the high and low weight-loss groups (159 OTUs belonging to 18 genera were significantly different, 101 of which were greater in the high compared to low weight-loss group; Additional File 12). A total of 48 OTUs belonging to 9 genera were found to be significantly different in pre-diet abundance between the low and mid weight-loss groups (Additional File 13), whilst 28 OTUs belonging to 6 genera were found to be significantly different between the mid and high weight-loss groups (Additional File 14).

### Microbiome structure as a predictor of weight loss

**Fig. 6:**
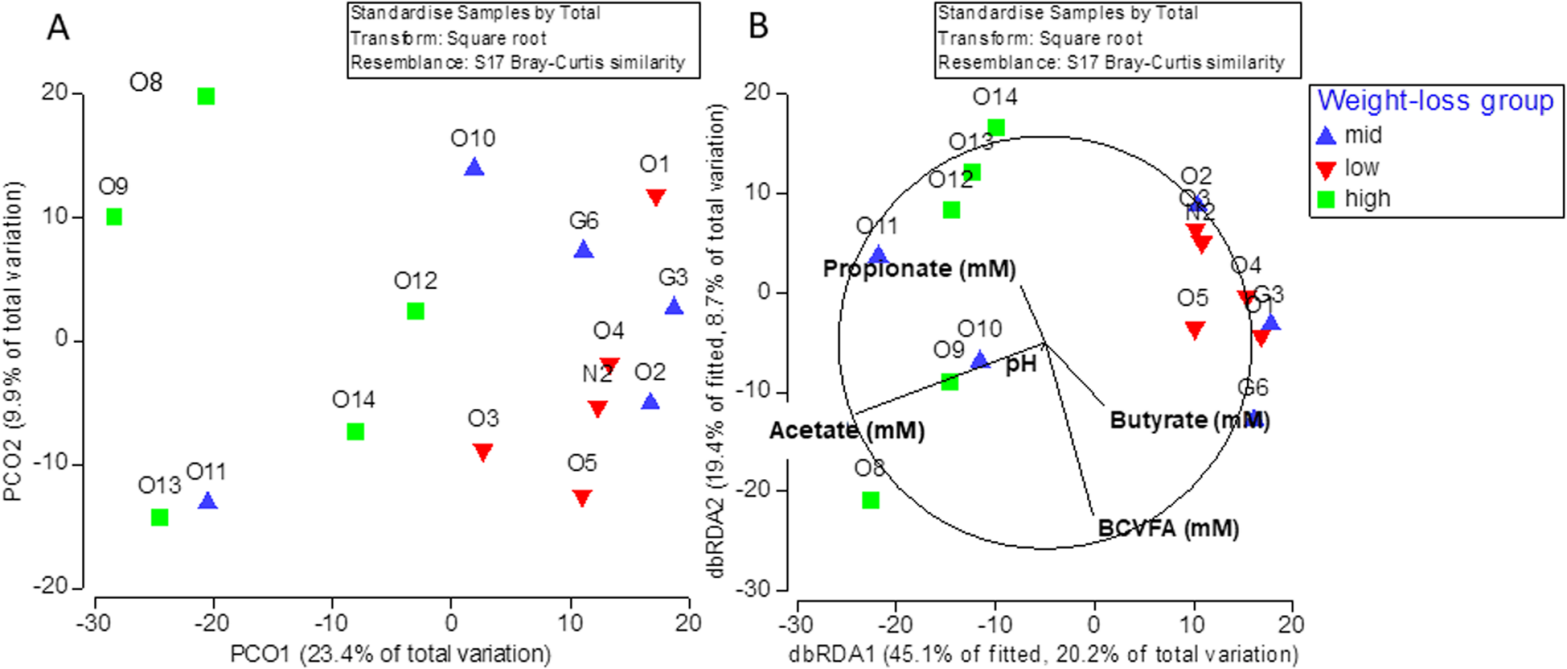
(A) Principal Co-ordinate analysis plot of the pre-diet bacterial community from the three weight-loss groupings using Bray-Curtis similarity index. (B) Distance-based redundancy analysis illustrating associations between the pre-diet bacterial community structure and faecal volatile fatty acid concentrations (mM) and pH

In an attempt to ascertain whether the pre-diet bacterial community structure could predict subsequent weight-loss, PERMANOVA and ANOSIM analysis was employed. There were significant differences in pre-diet bacterial community structure at the OTU level, whereby the greatest divergence was found between the high and low weight-loss groups (R = 0.67, *p* < 0.01; Table 8). A Principal Co-ordinate (PCO) analysis was performed using Bray-Curtis distance matrices to illustrate the clustering of the pre-diet bacterial community structure by weight-loss groupings (Fig. 6a). Whilst there was some overlapping between the groups, animals in the low weight-loss group clustered together and markedly separately from those in the high weight-loss group where there was more variation in pre-diet bacterial structure between animals. An additional PCO analysis was performed to evaluate associations between the post-diet bacterial community structure and weight-loss groups (Additional File 15) and shows similar clustering of animals as for the pre-diet bacterial community structure. Distance-based redundancy analysis (dbRDA) was performed to evaluate the contribution of pre-diet VFA concentrations and pH to pre-diet bacterial community structure (Fig. 6b). Pre-diet acetate concentration correlated strongly with differences in pre-diet bacterial community structure between the weight-loss groups.

**Table 8:** Summary of ANOSIM and PERMANOVA outputs

Group			
PERMANOVA (*p*-value)		Low	Medium
	Medium	0.41	
	High	**0.008**	0.06
ANOSIM (R-value)			
	Medium	0.028	
	High	0.668	0.316

Results matrices for pre-diet faecal bacterial community structure between weight-loss groups. Significant *P*-values for PERMANOVA (*P* < 0.05) are highlighted. Anosim R values indicate the degree of separation between samples (0 = very similar; 1 = highly dissimilar), with significant R-values (with *P* < 0.05) shown in bold.

## Discussion

To the authors’ knowledge, this is the first study to describe changes in the faecal microbiome following dietary restriction in ponies. The current study was repeated across two years to increase the number of animals involved, and therefore the statistical power in the subsequent data analysis. Unexpectedly, when animals were subsequently ranked according to their weight-loss, those that were recruited in the second year appeared to be more sensitive to weight-loss, and therefore the groupings appear to be confounded by year. However, the authors are confident that the results from the current study are a true effect of weight-loss and not a year effect as 13/15 of the animals from the current study were part of a larger study (*n* = 35 ponies) that was conducted over the same two years [8]. Findings from that study when animals were fed the same hay-based diet found no effect of year on the composition of the faecal microbiome, bacterial diversity or VFA concentrations [8].

The degree of weight-loss achieved during the dietary restriction phase of the current study was similar to that recorded in previous equine weight-loss studies [13, 33], whereby the greatest loss of BM occurred during the first week of dietary restriction, and thereafter weight-loss remained fairly constant. Despite the animals used in the current study being of the same breed and sex, there was still a considerable range in terms of weight-loss responsiveness, with an almost 2-fold difference in percentage weight-loss achieved between the animals losing the greatest and least amounts of weight. This would indicate that, as for humans, genetics may not be solely responsible for determining weight-loss responsiveness, and other biological factors are crucial in determining an individual animal’s propensity to weight-loss.

The relative contribution of fat mass (FM) and fat-free mass (FFM) to overall weight-loss has been extensively studied, and during dietary restriction in ponies, reductions in both FM and FFM have been observed [33]. It is generally accepted that across species, losses of FFM are lowest in individuals with a greater proportion of body fat at outset [34]. However, in the current study, a greater proportion of weight loss was compromised of lean mass compared to fat mass, and although there was a trend for increased loss of body fat in individuals with a greater outset body fat percentage, it did not reach statistical significance. The addition of exercise to weight-loss programs is often advocated to reduce losses of FFM [35, 36], and although ponies in the current study were allowed some exercise, greater amounts of exercise may have minimised the loss of lean mass.

As a significant portion of glucose uptake occurs in skeletal muscle relative to adipose tissue, the greater loss of FFM following weight-loss in the current study may have contributed to the increase in glucose clearance time and area under the curve for glucose observed in individual animals following the CGIT. It could also be suggested that the relatively short duration and degree of dietary restriction may have also contributed to the observed findings, as another equine weight-loss study of similar duration but a lower degree of dietary restriction also found no change in glucose dynamics following weight loss [37]. This is an area that will be analysed and discussed in greater depth in a planned companion paper.

The proportions of individual phyla recorded in the current study are in agreement with other equine microbiome studies utilising faecal samples, whereby *Bacteroidetes* and *Firmicutes* are dominant [26, 31, 32]. The current study identified a reduction in the proportion of the butyrate-producing phyla *Firmicutes* and *Tenericutes* following weight-loss. In humans, a reduction in *Firmicutes* and corresponding increase in the proportion of *Bacteroidetes* was identified following dietary restriction [38], restoring the ratio of *Firmicutes:Bacteroidetes* towards values obtained for lean humans. Whilst several studies have observed that a greater proportion of *Bacteroidetes* and reduced proportion of *Firmicutes* is associated with obesity [3, 39], others have found the opposite or no effect of obesity on the abundance of these two phyla [19, 20]. Although a relatively minor phylum in terms of relative abundance, *Actinobacteria* was found to be present in greater pre-diet abundance in the low compared to the high weight-loss groups in the current study. *Actinobacteria* appear to have multiple functional roles in gut metabolism and immunity [40], and has been shown to be reduced in relative abundance in human patients following gastric bypass, with outset proportions greater in those patients reaching diabetes remission [41]. Further studies would be required to determine the precise function of this bacterial phylum in the horse. At the genera level, whilst a large proportion of bacteria remain unclassified, the pre-diet abundance of *Mobilitalea* (*Lachnospiraceae* family) was found to be present in greater abundance in those animals in the low weight-loss compared to the high weight-loss group. Members of the *Lachnospiraceae* and *Ruminococcaceae* families have been identified to possess a greater number of genes associated with the ability to degrade a wide variety of polysaccharides and cleave cellulose and hemicellulose components of plant material [42]. It is tempting to speculate that the greater pre-diet abundance of *Mobilitalea* in those animals losing less overall body mass in the current study led to more efficient extraction of VFAs from the diet to supply energy requirements during a state of negative energy balance, thereby limiting the requirement to breakdown endogenous white adipose tissue stores of triglycerides. Further studies would be required to determine the precise function of *Mobilitalea* on VFA production and uptake in the horse.

In the current study, the reduction in measures of diversity following weight-loss and negative association between pre-diet diversity and subsequent weight-loss was an interesting finding. A high gut bacterial diversity is linked to greater overall metabolic and gastrointestinal health in humans [43, 44]. Additionally, a high gut diversity combined with a high fibre intake has been associated with lower long-term weight gain in humans [45]. However, a recent study identified that after 28 days of a high-fibre diet, there was a reduction in microbial gene richness and body weight, and an increase in the abundance of a select group of VFA-producing bacterial species that improved markers of glycaemic control [46]. The reduction in gene richness may be due to the enrichment of bacterial species that specifically digest dietary fibre. It could be suggested that although the nutrient composition of the diet remained the same during the weight-loss phase of the current study, the reduction in nutrient provision may have resulted the resident bacterial populations specialised in fibre fermentation out-competing some smaller bacterial populations in order to maximise energy extraction from the diet. This in turn would lead to the enrichment of fibre-digesting bacteria, reducing the overall species richness and diversity of bacteria residing within the gastrointestinal tract. The lower weight-loss responsiveness of ponies with higher pre-diet diversity in the current study is suggestive that a greater diversity results in resilience to changes in nutrient availability. This has been demonstrated in an ecological context, whereby greater species richness in soil resulted in reduced survival of an invader species [47]. Furthermore, human individuals with a low microbial gene richness were found to have higher adiposity, more pronounced insulin resistance and dyslipidaemia compared to individuals with a high microbial gene richness [23]. Additionally, genetically distinct bacterial populations may perform similar functional roles, and are therefore better able to adapt to changing environmental conditions, further supporting the current finding that increased pre-diet bacterial diversity resulted in less weight-loss [48].

As end products of microbial fermentation and key energy sources for the host, VFAs play crucial roles in cross-talking pathways linking the gut microbiota to energy balance. Humans are thought to obtain around 10% of their daily energy requirements from VFAs [49], whilst other mammals such as horses and ruminants are likely to utilise a greater proportion of VFAs as an energy source [50]. The fate of VFAs produced in the gastrointestinal tract by gut bacterial community varies for individual VFAs. In humans and mammals, a large portion of butyrate produced is utilised as the major energy substrate for colonocytes, and is therefore vital in maintaining gut health, whilst the remainder is transported to the liver to be metabolised, where propionate is also transported to be used as a substrate for gluconeogenesis [51]. Indeed, up to 60% of blood glucose has been found to derive from gluconeogenesis of propionate in ponies [52]. The most abundant VFA, acetate, is considered to be a major fuel in peripheral tissues of the horse, whereby plasma acetate levels were found to be greater for hay fed compared to grain-hay fed animals [53]. Additionally, acetate has also been found to be utilised preferentially over glucose for lipogenesis in vitro using equine adipose tissues [54]. Increased concentrations of total faecal VFA have been identified in obese and overweight human subjects compared to lean subjects [55], whilst plasma but not faecal acetate concentration was found to be greater in overweight compared to moderate condition mares [56].

The reduction in faecal VFA concentrations following weight-loss in the current study was unsurprising due to the reduction in dietary fibre intake during the weight-loss phase of the study (2% BM as DM daily during ‘pre-diet’ phase vs. 1% BM as DM daily during weight-loss phase). However, the strong positive associations between individual pre-diet faecal VFA concentrations (most notably acetate) and subsequent weight-loss was unexpected. Although it is impossible to speculate as to whether the reduced excretion of VFAs in those animals losing less weight is due to a reduced production and/or increased absorption, a human study identified that faecal acetate concentrations were inversely associated with acetate absorption from the rectum and distal colon [57]. Furthermore, a recent human study confirmed that acetate is primarily responsible for the inhibition of intracellular lipolysis in human multipotent adipose tissue-derived stem cells [58]. Therefore, one possible explanation for the lower weight-loss observed in animals with lower pre-diet faecal acetate concentrations could be that these animals are more metabolically efficient and have a greater production/absorption of VFAs into the circulation, inhibiting lipolysis and providing the host with a source of energy to limit the effects of the negative energy balance.

In support of this hypothesis, analysis of genera that differed in abundance between pre-diet acetate concentrations in the current study revealed that pre-diet abundance of *Sphaerochaeta* and *Treponema* (both belonging to the *Spirochaetes* phylum) were significantly greater in animals with the lowest faecal acetate concentration. The defining characteristic of the *Spirochaetes* phylum is their spiral shape and mobility due to the presence of an endoflagella [59]. However, the genus *Sphaerochaeta* has been found to lack this motility and has acquired an abundance of genes related to metabolism and fermentation from *Clostridia* [60]. Together, it could be suggested that animals losing less weight had a more diverse gut microbiome composition with a greater fermentative capacity and production/uptake of VFAs into the circulation to provide an energy source, limiting the breakdown of triglyceride stores during negative energy balance. Although fibre digestibility was unchanged following weight-loss in the current study, the positive association between the change in fibre digestibility and subsequent weight-loss was an interesting finding. It suggests that those animals losing less weight have a greater ability to increase fibre digestibility and thereby become more metabolically efficient during states of negative energy balance compared to those losing more weight.

Evaluation of pre-diet microbiome community structure by dbRDA by weight-loss group revealed clustering of animals within their weight-loss grouping, a finding that became more pronounced when pre-diet VFA concentrations were included in the analysis. In combination with the high ROC AUC and sensitivity/specificity for pre-diet acetate in predicting subsequent weight-loss, these data would suggest that future weight-loss studies using animals of different breeds and gender should evaluate pre-diet faecal acetate as a potential marker of weight-loss sensitivity.

A limitation of the current study and with all 16S metataxonomic-based studies is the lack of bacterial functional information generated. Additionally, methodological variation arising from the range in sequencing platforms, pipelines and primers utilised by different research groups will influence the resulting sequence data obtained, making it difficult to make direct comparisons between similar studies. Finally, although animals were allowed pasture turnout on a daily basis in the current study, it was out-with the scope of the current study to quantify the energy expended during this turnout period. However, it is likely that this would be negligible in comparison to the restricted energy provision of the diet as animals appeared to spend the majority of the turnout time walking, rolling and grooming each other. Future studies should be directed towards understanding the functional capacity of the bacterial community and potential metabolic pathways influenced by bacterial community structure following weight-loss.

## Conclusions

The current study is the first to describe changes in the faecal bacterial community following a period of weight-loss in ponies. There were significant changes in the microbiome composition, diversity and faecal VFA concentrations following weight-loss. Additionally, measures of pre-diet diversity, VFA concentration and bacterial community structure were all significantly associated with subsequent weight-loss. This data will form the basis for further research evaluating the potential of faecal VFAs and elements of the faecal microbiome in predicting subsequent weight-loss in horses and ponies.

## Methods

### Animals and husbandry

Sixteen obese (BCS > 7/9), Welsh Mountain (Section A) pony mares (*n* = 8 Year 1; n = 8 Year 2), were studied over the same 11-week period (December–February; Table 2) in both years. The animals were part of a larger study [8], evaluating the impact of obesity, age and diet on the faecal microbiome and were loaned to the study by local pony breeders within a 30-mile radius of the University of Liverpool’s, Ness Heath Farm. All animals were clinically examined at the point of recruitment, and were considered to be in good overall health. Routine foot care, vaccination and anthelmintic protocols were maintained throughout the study. Animals were individually housed in (3 m × 5 m) loose boxes within the same barn, bedded on wood shavings and had free access to fresh water at all times. When possible, ponies were given access to pasture for 30 min daily to permit basal exercise and social contact. To ensure total control over nutrition, which was provided within the stables, pasture intake while at exercise was prevented using closed grazing muzzles (Shires).

All procedures were conducted in accordance with Home Office (ASPA) requirements, approved by the University of Liverpool’s Animal Welfare Ethics Committee (Home Office project license number: PPL 70/8475). Written informed consent was obtained from all owners. Upon completion of the study, all animals were returned to their owners care.

### Study design

This 11-week study was designed to allow the evaluation of any associations between weight loss responsiveness and changes in the gastro-intestinal microbiome during a 7-week period of dietary restriction. Animals were maintained on a grass hay diet (same batch across both years) throughout, as for [8].

Prior to the initiation of negative energy balance, animals first underwent a 4-week period of adaptation to the study diet and husbandry conditions. During this 4-week ‘pre-diet’ period, individual animals were offered grass hay at 2% of their outset BM as DM daily, a plane of nutrition estimated to approximate maintenance in terms of digestible energy (DE) and crude protein (CP) content. The DE value of the hay diet was retrospectively calculated from the pre-diet digestibility trial and was found to average 8.21 ± 0.95 MJ/kg BM. Therefore, the provision of 2% BM as DM hay daily during the pre-diet phase was calculated to provide an average of 117.82 ± 13.67% of estimated maintenance requirements based on NRC recommendations of 33.3 kcal/kg BM/day [61]. Based on the crude protein (CP) content of the hay and balancer, the pre-diet phase was found to provide 147.50% of estimated maintenance requirements based on NRC recommendations of 1.26 g CP/kg BM/day [61].

To mimic standard husbandry practices, daily hay allowances for each animal were equally divided and offered as two daily meals (08.30 h and 16.30 h). To ensure adequacy of vitamin and mineral intakes, a moistened nutrient balancer product (Spillers Lite) was fed daily (08.30 h, 0.1% BM as DM). Mean compositions for key nutrients were: Hay; DM 88.6%, Gross energy (GE) 18.9 MJ/kg DM, Ash 4.0%, CP 8.1%, Acid detergent fibre (ADF) 41.2%, Neutral detergent fibre (NDF) 64.7%, starch 0.6%, water soluble carbohydrates (WSC) 15.6%. Vitamin / mineral balancer; DM 93.0%, GE 16.35 MJ/kg DM, DE 9.5 MJ/kg DM (estimation provided by manufacturer), Ash 14.0%, CP 21.1%, ADF 14.3%, NDF 31.7%, starch 10.5%, WSC 12.5%. Samples of hay and balancer were analysed by a commercial laboratory utilising wet chemistry procedures (Dairy One, Ithaca, USA), prior to the 4-week pre-diet phase in both years and there was no difference in the composition of key nutrients between years.

For the remaining seven weeks of the study, hay intake was restricted to 1% BM as DM daily with 0.1% BM as a nutrient balancer meal. Individual feed provisions were recalculated weekly during this period to account for changes in BM. This degree of negative energy balance was predicted to induce a mean weight loss of 1% of outset BM weekly, and based on calculated DE values for the hay diet from the pre-diet digestibility trial, this degree of dietary restriction provided individual animals with an average of 58.91 ± 6.84% of estimated DE requirements [61].

### Physical measurements

Each week, BM was recorded (to nearest 500 g) between 08.30 h and 09.00 h (regularly calibrated weighbridge: Lightweight Intermediate; Horseweigh), before receiving their morning hay provision. BCS was recorded for each animal on a weekly basis using the Kohnke (1992) modification of the Henneke et al. (1983) system, by the same individual throughout the duration of the study.

### Faecal collection

Faecal samples were collected across the final 3 consecutive days of the ‘pre-diet’ period and again during the final three consecutive days of dietary restriction. Samples of faeces were collected as previously described [8]. In brief, faeces were sampled from each animal into a clean steel bowl within 5 min of the first defecation spontaneously voided after 09.00. Samples were then hand-mixed and aliquoted into four, 5 ml sterile vials (Scientific Laboratory Supplies, UK), snap-frozen in liquid nitrogen and stored at − 80 °C prior to DNA extraction.

### Estimates of total body composition

Total body water (TBW) and total body fat mass were calculated twice for each individual animal; once during the final week of the ‘pre-diet’ phase and again during the final week of the dietary restriction phase, using the deuterium oxide (D_2_O) dilution method, as previously described and validated for clinical use in the pony [62]. Deuterium enrichments in plasma samples were analysed as previously described [8].

### Combined glucose- insulin tolerance test (CGIT)

A dynamic CGIT test was performed twice for each individual animal; once during the final week of the ‘pre-diet’ phase and again during the final week of the dietary restriction [13]. Blood samples were collected, processed and analysed for glucose and insulin concentrations as previously described [8],

### Apparent digestibility

Digestibility trials were conducted twice for individual ponies by total faecal collection over 3 consecutive days (72 h) once during the final week of the ‘pre-diet’ phase and again during the final week of the dietary restriction. Across the same 72 h, any refused feed was weighed and recorded at the end of each 24-h period. Total daily faecal collections were weighed, thoroughly mixed and duplicate samples were collected for analysis. Dry matter (DM), ash, and GE content (MJ/kg DM) of faeces were determined as previously described [8]. Additionally, the NDF content of the dried faecal samples was measured using the ANKOM Technology method at a commercial laboratory (Dairy One, Ithaca, USA).

### DNA extraction and ion torrent next generation sequencing

Faecal samples were subjected to DNA extraction and Ion Torrent next generation sequencing methods, as previously described [8].

Following sequencing, data were processed as previously described [8]. Briefly, sample identification numbers were assigned to multiplexed reads using the MOTHUR software environment. Data were denoised by removing low-quality sequences, sequencing errors and chimeras (quality parameters: maximum 10 homopolymers, qaverage 13, qwindow 25, for archaea the qwindow was set at 30, and erate = 1; Chimera check, both de novo and database driven using Uchime). Sequences were clustered into OTUs using the Uparse pipeline at 97% identity. Bacterial taxonomic information on 16S rRNA sequences was obtained by comparing against Ribosomal Database Project-II [63]. The number of reads per sample were normalised to the sample with the lowest number of sequences.

### Volatile fatty acid measurement

Concentrations of volatile fatty acids in faecal samples were analysed by gas liquid chromatography as previously described [8]. Resultant VFA concentrations were adjusted to the dry matter content of the faecal samples.

### Statistical analysis

All data was inputted into Excel and exported for statistical analysis into STATA 13.1 (Statacorp, Texas). The pattern of weight change during the weight-loss phase of the study was investigated by fitting a mixed effects regression model with body mass (kg) as the outcome variable. Outset body mass (Week 0) was included to account for differences between individual animals in outset BM. Time (week) was added as an explanatory variable and polynomial terms were fitted if they improved model fit as judged by the likelihood ratio test. Pony identity was included as a random intercept with time as a random slope. An unstructured covariance matrix was employed for the random effects. Body mass was predicted from the model and using this, a proportional weight loss (adjusted to Week 0) was calculated and used to rank animals according to weight-loss. This was then divided into terciles (high, mid and low weight-loss) to enable further interrogation of the data. Wilcoxon sign-rank test was employed to evaluate differences in digestibility, body composition and glucose/insulin dynamics between pre- and post-diet measurements.

Diversity indices (Inverse Simpson’s and Shannon-Wiener), species observed (S.Obs) and estimated species richness (S.Chao1) were calculated for all faecal samples at all time points using normalized data as recommended to reduce over-inflation of true diversity in pyrosequencing data sets [64]. Data were assessed for normality visually adopting the “ladder of powers” approach. Transformations were performed where appropriate. Student t tests were performed to assess mean changes in VFA concentrations and diversity indices between the pre-diet and post-diet period (using arithmetic mean of final 3 days of pre-diet and post-diet samples). One-way ANOVA (Genstat® 12th edition; VSN International ltd.) was employed to assess differences in pre-diet diversity and faecal VFA concentrations between weight-loss groups (high, mid, low), using Bonferroni-adjusted *P* values to correct for multiple testing. *P*-values were considered significant if < 0.05. Findings were confirmed by univariate analysis (STATA 13.1, Statcorp, Texas) whereby pre-diet diversity or faecal VFA concentration was the explanatory variable and overall proportional weight-loss (logit transformed) was the outcome variable. In order to investigate further the ability of pre-diet acetate concentration to predict the weight-loss success of an animal, a binary (yes/no) variable was generated whereby weight-loss success was defined as total percentage weight-loss of ≥8%. This value was selected on the basis of data collected within the current study. A receiver operating characteristic (ROC) curve was generated for pre-diet acetate concentration as a predictor of weight-loss (yes/no). This allowed investigation of the optimal cut-off score for pre-diet acetate concentration and generated sensitivity and specificity parameters. To investigate differences in the pre-diet relative abundance of individual bacterial genera between concentrations of pre-diet acetate (mean of 3 pre-diet days), acetate concentrations were divided into quartiles as follows: Quartile 1, *n* = 4, 11.45 mM ± 0.61 (mean ± SD); Quartile 2, n = 4, 13.93 mM ± 1.37, Quartile 3, *n* = 3, 20.69 mM ± 1.95, Quartile 4, n = 4, 28.77 mM ± 5.67 and data were subjected to ANOVA analysis (Genstat® 12th edition; VSN International ltd.). Statistical analyses excluded those for which an abundance of less than 0.05% was recorded. *P* values were adjusted for multiple testing using the method proposed by Benjamini and Hochberg [65] to decrease the false discovery rate. Findings with *P* < 0.10 when applying Benjamini and Hochberg (1995) correction were regarded as statistically significant.

Phylum and genera level differences in the microbiome following weight-loss and between weight-loss groups were investigated by ANOVA as described above.

Differential abundances at an OTU level were evaluated using the bioconductor package DESEQ21 in the statistical package R, a methodology appropriate for the interrogation of high-throughput, sequencing count data, allowing models to be built using a negative binomial distribution to account for the distribution of read counts from each OTU [66].

Permutation multivariate analysis of variance (PERMANOVA) was used to determine overall significant differences in bacterial communities. Analyses were performed in PRIMER 6 & PERMANOVA+ (versions 6.1.18 and 1.0.8 respectively; Primer-E, Ivybridge, UK). Abundance percentage data were subjected to square-root transformation and Bray-Curtis distance matrices were calculated. PERMANOVA was carried out using default settings with 9999 unrestricted permutations and the Monte Carlo P value was calculated. Analyses of Similarity (ANOSIM) were conducted in PRIMER 6 & PERMANOVA+, using the Bray-Curtis distance matrix calculated above. This analysis was used to provide a metric of the degree of divergence between communities, as given by the R statistic.

To calculate the contribution of environmental data on bacterial communities, distance-based linear modelling was used to calculate which environmental variables had a significant correlation with the community data. Significant variables were used in distance-based redundancy analysis (dbRDA) [67] as implemented in PRIMER 6 and PERMANOVA+.

## Supplementary information

**Additional File 1.** Associations between weight-loss and outset/change in digestibility.

**Additional File 2 **Phylogenetic tree depicting clustering of the faecal bacterial microbiome within weight-loss phase and animal (*n* = 15).

**Additional File 3.** Rarefaction curves.

**Additional File 4.** Associations between weight-loss and outset measures of diversity.

**Additional File 5.** Associations between weight-loss and outset VFA concentrations.

**Additional File 6.** Receiver operating characteristic (ROC) curve for outset acetate concentration as a predictor of animals achieving ≥8% weight-loss. At the optimal cut-off of 14 mM acetate, sensitivity was 88.9%, while specificity was 88.3%.

**Additional File 7.** Relative abundance of outset bacterial genera (mean of 3 pre-diet days) between animals grouped into quartiles by outset acetate concentrations (mean of 3 pre-diet days).

**Additional File 8 **Relative abundance of pre-diet bacterial phyla (mean of 3 pre-diet days) between the three weight-loss groups (*n* = 5/group).

**Additional File 9.** Classification of the 33 bacterial families unclassified at the genera level.

**Additional File 10.** Relative abundance of outset bacterial genera (mean of 3 pre-diet days) between the three weight-loss groups (n = 5/group).

**Additional File 11.** Relative abundance of bacterial OTUs significantly different in abundance before (pre-diet), and after 7 weeks of dietary restriction (post-diet; n = 15).

**Additional File 12.** Relative abundance of outset bacterial OTUs significantly different in abundance between high and low weight-loss groups (n = 5/group).

**Additional File 13.** Relative abundance of outset bacterial OTUs significantly different in abundance between low and mid weight-loss groups (n = 5/group).

**Additional File 14.** Relative abundance of outset bacterial OTUs significantly different in abundance between mid and high weight-loss groups (n = 5/group).

**Additional File 15.** Principal Co-ordinate analysis plot of the post-diet bacterial community from the three weight-loss groupings using Bray-Curtis similarity index.

## Data Availability

Raw sequences reads from the bacterial libraries were deposited at the EBI Short Read Archive from of the European Nucleotide Archive (accession number PRJEB34659).

## Abbreviations

ADF: Acid detergent fibre
AUC: Area under the curve
BCVFA: Branched-chain volatile fatty acids
BM: Body mass
CGIT: Combined glucose-insulin test
CP: Crude protein
DE: Digestible energy
DM: Dry matter
EMS: Equine metabolic syndrome
FFM: Fat-free mass
FM: Fat mass
GE: Gross energy
MJ: Megajoule
NDF: Neutral detergent fibre
NGS: Next-generation sequencing
OTU: Operational taxonomic unit
PCO: Principal coordinate analysis
ROC: Receiver operating characteristic
TBW: Total body water
VFA: Volatile fatty acids
WSC: Water soluble carbohydrate
