# Supplementary material for: The equine gastrointestinal microbiome: impacts of weight-loss
Source: BMC Vet Res. 2020 Mar 4;16:78. doi: 10.1186/s12917-020-02295-6 (PMC7057583; doi:10.1186/s12917-020-02295-6)
Supplement: Supplementary file 1 — Additional File 1. Associations between weight-loss and outset/change in digestibility. [file 12917_2020_2295_MOESM1_ESM.pdf]

**Additional File 1**: Associations between weight-loss and outset/change in digestibility.

| Explanatory variable | Coefficient | 95% CI | P value | R-squared |
|---|---|---|---|---|
| Outset GE digestibility | 0.02 | 0.002 to 0.04 | 0.04 | 0.24 |
| Baseline | -3.41 | -4.37 to -2.45 | < 0.01 | |
| Outset DM digestibility | 0.02 | 0.001 to 0.05 | 0.04 | 0.23 |
| Baseline | -3.62 | -4.79 to -2.44 | < 0.01 | |
| Outset NDF digestibility | 0.02 | 0.004 to 0.033 | 0.02 | 0.33 |
| Baseline | -3.30 | -4.03 to -2.58 | < 0.01 | |
| Change in GE digestibility | 0.02 | 0.001 to 0.034 | 0.04 | 0.24 |
| Baseline | -2.35 | -2.46 to -2.24 | < 0.01 | |
| Change in DM digestibility | 0.02 | -0.0002 to 0.035 | 0.05 | 0.20 |
| Baseline | -2.34 | -2.46 to -2.23 | < 0.01 | |
| Change in NDF digestibility | 0.02 | 0.003 to 0.036 | 0.03 | 0.28 |
| Baseline | -2.37 | -2.48 to -2.27 | < 0.01 | |

Univariate regression analysis was employed to investigate associations between total proportional weight-loss (corrected to week 0; logit transformation) and outset digestibility (DM, GE, NDF) and the change in digestibility (pre-diet minus post-diet) following weight-loss (DM, GE, NDF).
