# Supplementary material for: The equine gastrointestinal microbiome: impacts of weight-loss
Source: BMC Vet Res. 2020 Mar 4;16:78. doi: 10.1186/s12917-020-02295-6 (PMC7057583; doi:10.1186/s12917-020-02295-6)

Additional File 2. Phylogenetic tree depicting clustering of the faecal bacterial microbiome within weight-loss phase and animal (n = 15).

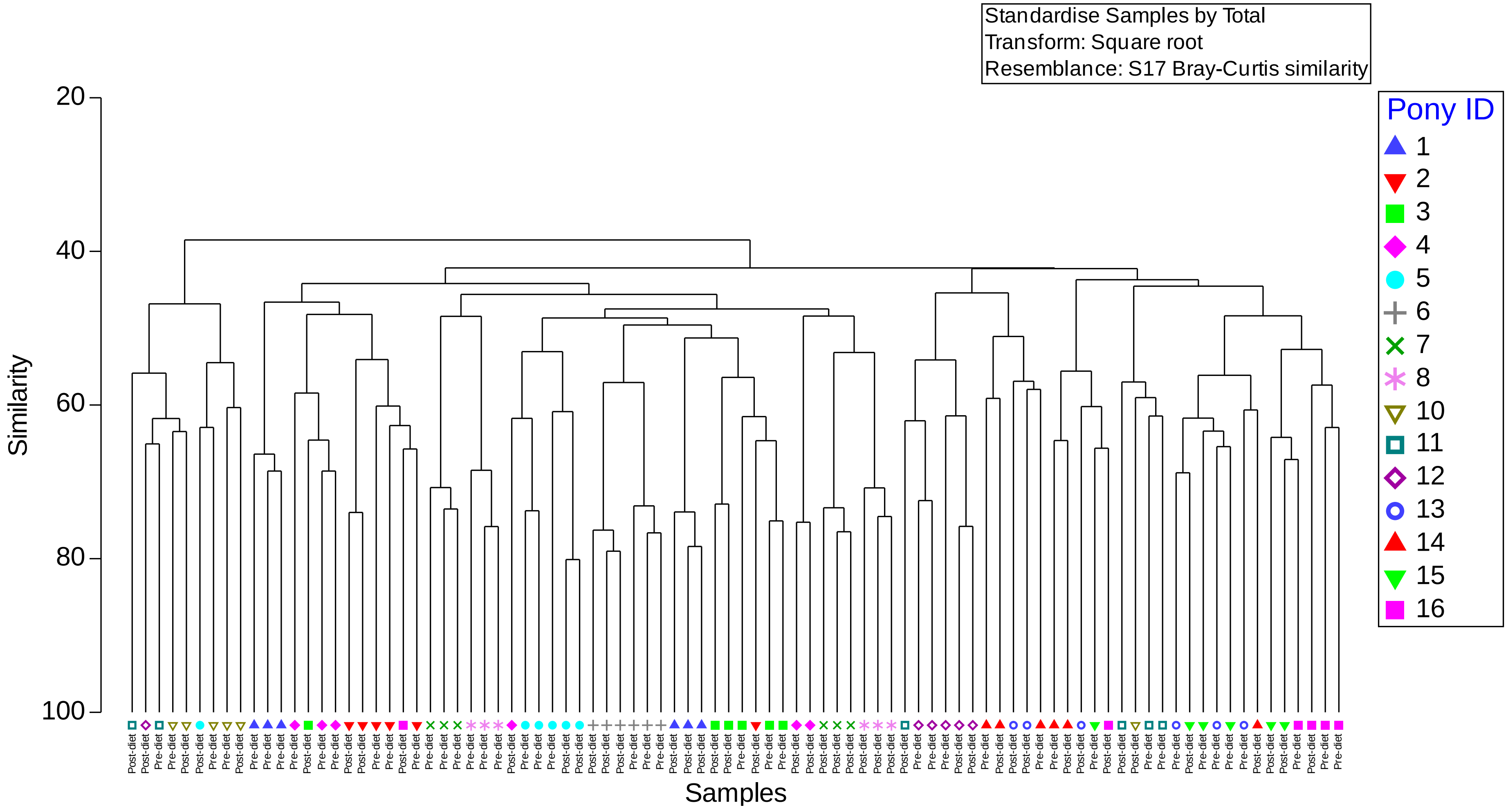

Supplement: Supplementary file 2 — Additional File 2 Phylogenetic tree depicting clustering of the faecal bacterial microbiome within weight-loss phase and animal (n = 15). [file 12917_2020_2295_MOESM2_ESM.pdf]
