# Supplementary figures and images for: The equine gastrointestinal microbiome: impacts of weight-loss
Source: BMC Vet Res. 2020 Mar 4;16:78. doi: 10.1186/s12917-020-02295-6 (PMC7057583; doi:10.1186/s12917-020-02295-6)

**Additional File 3.** Rarefaction curves

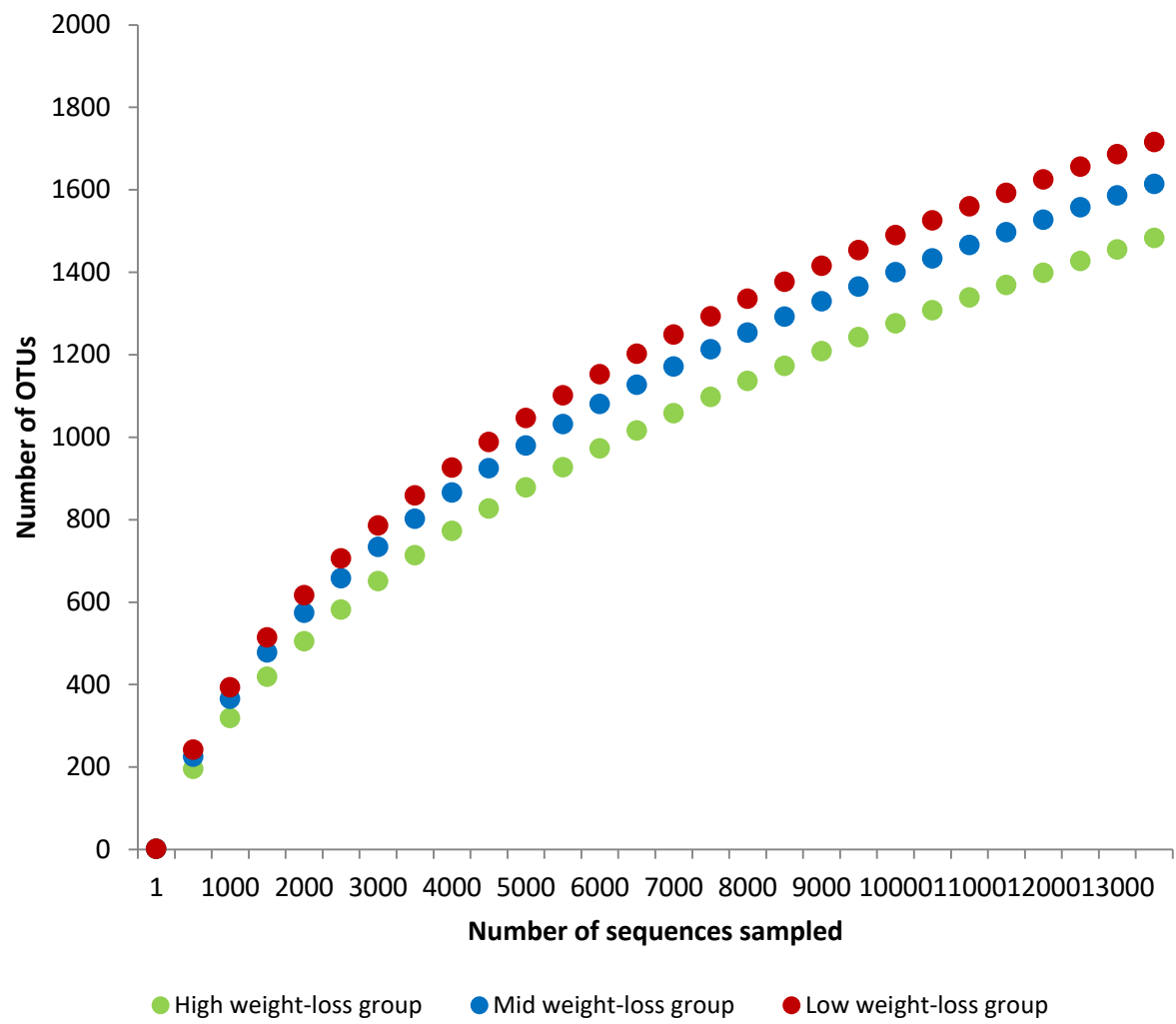

Supplement: Supplementary file 3 — Additional File 3. Rarefaction curves. [file 12917_2020_2295_MOESM3_ESM.pdf]
