# Supplementary material for: The equine gastrointestinal microbiome: impacts of weight-loss
Source: BMC Vet Res. 2020 Mar 4;16:78. doi: 10.1186/s12917-020-02295-6 (PMC7057583; doi:10.1186/s12917-020-02295-6)
Supplement: Supplementary file 4 — Additional File 4. Associations between weight-loss and outset measures of diversity. [file 12917_2020_2295_MOESM4_ESM.pdf]

Additional File 4. Associations between weight-loss and outset measures of diversity.

Explanatory variable	Coefficient	95% CI	P value	R-squared
Inverse Simpson (logit transformation)	-0.002	-0.004 to 0.0003	0.10	0.20
Baseline	-2.25	-2.40 to -2.10	< 0.01	
Shannon-Weiner (squared transformation)	-0.02	-0.03 to -0.004	0.02	0.35
Baseline	-1.77	-2.25 to -1.29	< 0.01	
S.Obs	-0.0005	-0.0009 to -0.0001	0.01	0.39
Baseline	-1.50	-2.15 to -0.85	< 0.01	

Univariate regression analysis was employed to investigate associations with total proportional weight-loss (corrected to week 0; logit transformation) as the outcome variable and outset diversity measure (mean of 3 pre-diet days) as the explanatory variable.
