# Supplementary material for: The equine gastrointestinal microbiome: impacts of weight-loss
Source: BMC Vet Res. 2020 Mar 4;16:78. doi: 10.1186/s12917-020-02295-6 (PMC7057583; doi:10.1186/s12917-020-02295-6)
Supplement: Supplementary file 5 — Additional File 5. Associations between weight-loss and outset VFA concentrations. [file 12917_2020_2295_MOESM5_ESM.pdf]

Additional File 5. Associations between weight-loss and outset VFA concentrations.

Outcome variable	Explanatory variable	Coefficient	95% CI	P value	R-squared
Total proportional weight-loss	Outset acetate concentration	0.03	0.01 to 0.04	<0.01	0.52
	Baseline	-2.79	-3.05 to -2.53	<0.01	
	Outset butyrate concentration	0.29	0.05 to 0.54	0.02	0.34
	Baseline	-2.80	-3.18 to -2.43	<0.01	
	Outset propionate concentration	0.12	0.02 to 0.22	0.03	0.33
	Baseline	-2.88	-3.33 to -2.44	<0.01	
	Outset BCVFA concentration	0.16	-0.11 to 0.43	0.22	0.12
	Baseline	-2.57	-2.91 to -2.22	<0.01	

Univariate regression analysis was employed to investigate associations between total proportional weight-loss (corrected to week 0; logit transformation) and outset VFA concentrations (mean of 3 pre-diet days).
