# Supplementary material for: The equine gastrointestinal microbiome: impacts of weight-loss
Source: BMC Vet Res. 2020 Mar 4;16:78. doi: 10.1186/s12917-020-02295-6 (PMC7057583; doi:10.1186/s12917-020-02295-6)

Additional File 6. Receiver operating characteristic (ROC) curve for outset acetate concentration as a predictor of animals achieving ≥ 8% weight-loss. At the optimal cut-off of 14mM acetate, sensitivity was 88.9%, while specificity was 88.3%.

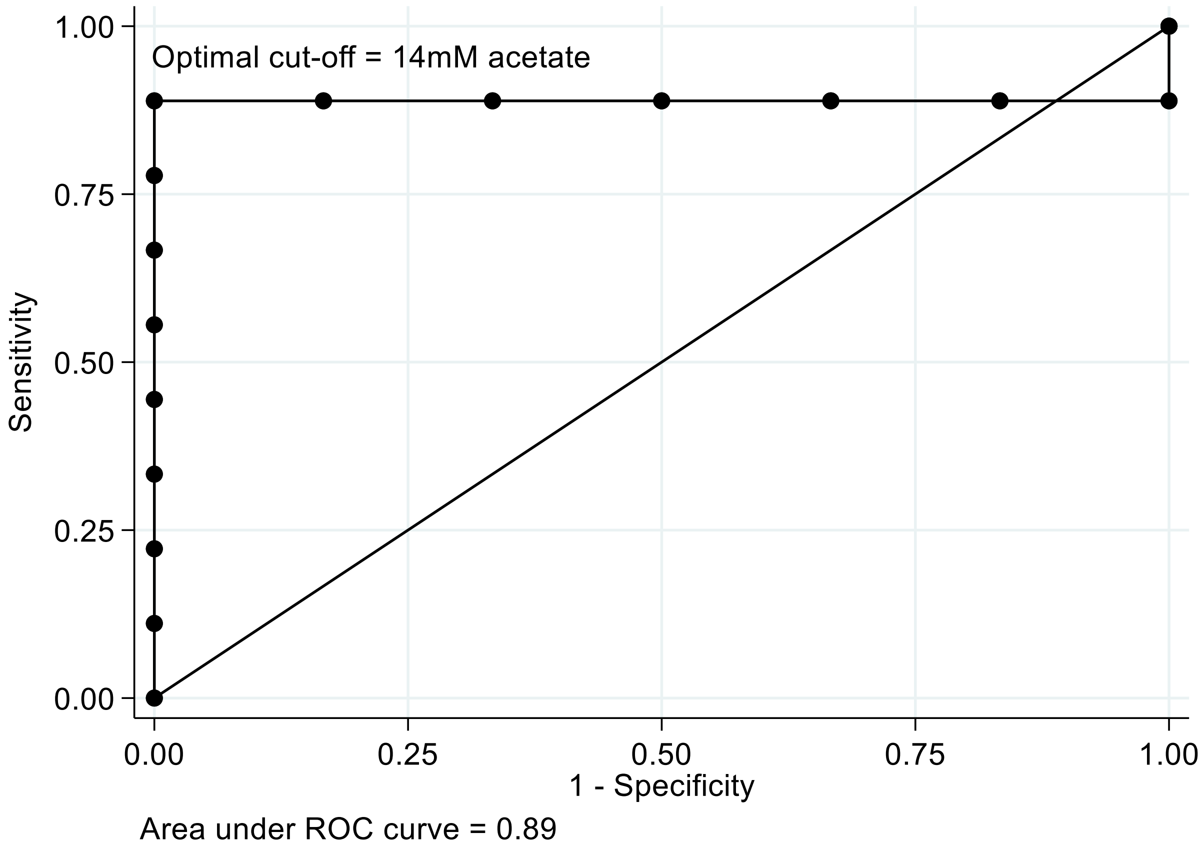

Supplement: Supplementary file 6 — Additional File 6. Receiver operating characteristic (ROC) curve for outset acetate concentration as a predictor of animals achieving ≥8% weight-loss. At the optimal cut-off of 14 mM acetate, sensitivity was 88.9%, while specificity was 88.3%. [file 12917_2020_2295_MOESM6_ESM.pdf]
