# Supplementary material for: The equine gastrointestinal microbiome: impacts of weight-loss
Source: BMC Vet Res. 2020 Mar 4;16:78. doi: 10.1186/s12917-020-02295-6 (PMC7057583; doi:10.1186/s12917-020-02295-6)
Supplement: Supplementary file 7 — Additional File 7. Relative abundance of outset bacterial genera (mean of 3 pre-diet days) between animals grouped into quartiles by outset acetate concentrations (mean of 3 pre-diet days). [file 12917_2020_2295_MOESM7_ESM.pdf]

Additional File 7. Relative abundance of outset bacterial genera (mean of 3 pre-diet days) between animals grouped into quartiles by outset acetate concentrations (mean of 3 pre-diet days).

	Quartiles of outset Acetate concentration				SED	Benjamini-Hochberg P-value
	1	2	3	4		
Unclassified	0.690	0.661	0.549	0.596	0.039	0.376
Fibrobacter	0.080	0.110	0.287	0.201	0.077	0.133
Treponema	0.037	0.038	0.015	0.018	0.021	0.051
Phascolarctobacterium	0.027	0.023	0.014	0.010	0.021	0.133
Alkalitalea	0.027	0.019	0.035	0.002	0.082	0.620
Prevotella	0.016	0.012	0.020	0.016	0.039	0.992
Paraprevotella	0.012	0.012	0.008	0.021	0.021	0.404
Paludibacter	0.012	0.013	0.007	0.029	0.050	0.676
Alloprevotella	0.010	0.004	0.001	0.003	0.028	0.453
Ruminococcus	0.010	0.013	0.007	0.010	0.024	0.870
Oscillibacter	0.009	0.007	0.005	0.006	0.012	0.423
Barnesiella	0.008	0.009	0.003	0.032	0.055	0.542
Clostridium XIVa	0.007	0.007	0.006	0.004	0.013	0.423
Lachnospiracea	0.005	0.005	0.004	0.005	0.009	0.978
Rikenella	0.005	0.009	0.006	0.008	0.013	0.433
Phocaeicola	0.004	0.014	0.008	0.009	0.028	0.432
Anaeroplasma	0.004	0.007	0.004	0.003	0.015	0.423
Faecalitalea	0.003	0.003	0.001	0.002	0.010	0.115
Asteroleplasma	0.003	0.001	0.002	0.001	0.021	0.870
Macellibacteroides	0.003	0.000	0.000	0.000	0.023	0.579
Anaerovorax	0.003	0.002	0.001	0.001	0.004	0.027
Sphaerochaeta	0.002	0.002	0.000	0.000	0.006	0.021
Coprobacter	0.002	0.001	0.001	0.002	0.016	0.978
Pseudoflavonifractor	0.002	0.011	0.001	0.004	0.039	0.870
Sporobacter	0.002	0.002	0.001	0.002	0.005	0.316
Lachnobacterium	0.002	0.001	0.002	0.001	0.010	0.870
Intestinimonas	0.002	0.002	0.002	0.003	0.010	0.752
Clostridium IV	0.002	0.002	0.002	0.002	0.067	0.978
Faecalicoccus	0.001	0.001	0.000	0.000	0.007	0.133
Mobilitalea	0.001	0.001	0.000	0.000	0.005	0.051
Anaerorhabdus	0.001	0.001	0.001	0.000	0.010	0.376
Saccharibacteria	0.001	0.001	0.001	0.001	0.007	0.997
Anaerocella	0.001	0.001	0.001	0.000	0.007	0.455
Ethanoligenens	0.001	0.000	0.001	0.001	0.010	0.870
Roseburia	0.001	0.001	0.001	0.001	0.005	0.997
Mogibacterium	0.001	0.001	0.000	0.001	0.006	0.453
Saccharofermentans	0.001	0.001	0.001	0.000	0.007	0.752
Vampirovibrio	0.001	0.001	0.001	0.001	0.007	0.870
Candidatusendomicrobium	0.000	0.001	0.000	0.000	0.010	0.978
Streptococcus	0.000	0.000	0.001	0.001	0.010	0.316

Catabacter	0.000	0.000	0.000	0.000	0.005	0.978

Animals were split into quartiles depending on outset acetate concentrations as follows: Quartile 1, n = 4, 9.45mM ± 0.27 (mean ± SD); Quartile 2, n = 4, 11.66mM ± 1.04, Quartile 3, n = 3, 16.76mM ± 1.13, Quartile 4, n = 4, 22.63mM ± 4.49. ANOVA analysis was employed to evaluate group differences in the relative abundance of bacterial genera, and the resulting P-values were adjusted for multiple testing using the Benjamini-Hochberg correction.
