# Supplementary material for: The equine gastrointestinal microbiome: impacts of weight-loss
Source: BMC Vet Res. 2020 Mar 4;16:78. doi: 10.1186/s12917-020-02295-6 (PMC7057583; doi:10.1186/s12917-020-02295-6)
Supplement: Supplementary file 8 — Additional File 8 Relative abundance of pre-diet bacterial phyla (mean of 3 pre-diet days) between the three weight-loss groups (n = 5/group). [file 12917_2020_2295_MOESM8_ESM.pdf]

Additional File 8. Relative abundance of pre-diet bacterial phyla (mean of 3 pre-diet days) between the three weight-loss groups (n = 5/group).

	**Low**	**Mid**	**High**	**SED**	**Benjamini-Hochberg P-value**
Firmicutes	0.493	0.476	0.493	0.028	0.156
Bacteroidetes	0.082	0.180	0.219	0.037	0.949
Fibrobacteres	0.279	0.230	0.217	0.07	0.156
Unclassified	0.058	0.049	0.034	0.024	0.156
Spirochaetes	0.053	0.042	0.022	0.021	0.033
Proteobacteria	0.020	0.016	0.009	0.016	0.132
Tenericutes	0.009	0.005	0.005	0.014	0.156
Verrucomicrobia	0.001	0.001	0.001	0.007	0.909
Candidatus Saccharibacteria	0.003	0.002	0.001	0.005	0.890
Actinobacteria	0.001	0.000	0.001	0.006	0.011
Elusimicrobia	0.000	0.000	0.000	0.008	0.909

ANOVA analysis was employed to evaluate group differences in the relative abundance of bacterial phyla, and the resulting P-values were adjusted for multiple testing using the Benjamini-Hochberg correction.
