# Supplementary material for: The equine gastrointestinal microbiome: impacts of weight-loss
Source: BMC Vet Res. 2020 Mar 4;16:78. doi: 10.1186/s12917-020-02295-6 (PMC7057583; doi:10.1186/s12917-020-02295-6)
Supplement: Supplementary file 9 — Additional File 9. Classification of the 33 bacterial families unclassified at the genera level. [file 12917_2020_2295_MOESM9_ESM.pdf]

Additional File 9. Classification of the 33 bacterial families unclassified at the genera level.

Phylum	Class	Order	Family
Firmicutes	Negativicutes	Selenomonadales	Acidaminococcaceae
Actinobacteria	Actinobacteria	Actinomycetales	Actinomycetaceae
Proteobacteria	Betaproteobacteria	Burkholderiales	Alcaligenaceae
Chloroflexi	Anaerolineae	Anaerolineales	Anaerolineaceae
Firmicutes	Bacilli	Bacillales	Bacillaceae 2
Firmicutes	Clostridia	Clostridiales	Clostridiaceae 4
Firmicutes	Clostridia	Clostridiales	Clostridiales_Incertae Sedis IV
Firmicutes	Clostridia	Clostridiales	Clostridiales_Incertae Sedis XII
Firmicutes	Clostridia	Clostridiales	Clostridiales_Incertae Sedis XIII
Actinobacteria	Actinobacteria	Coriobacteriales	Coriobacteriaceae
Proteobacteria	Deltaproteobacteria	Desulfovibrionales	Desulfovibrionaceae
Firmicutes	Erysipelotrichia	Erysipelotrichales	Erysipelotrichaceae
Firmicutes	Clostridia	Clostridiales	Eubacteriaceae
Bacteroidetes	Flavobacteriia	Flavobacteriales	Flavobacteriaceae
Firmicutes	Clostridia	Clostridiales	Lachnospiraceae
Bacteroidetes	Bacteroidia	Bacteroidales	Marinilabiliaceae
Actinobacteria	Actinobacteria	Actinomycetales	Microbacteriaceae
Tenericutes	Mollicutes	Mycoplasmatales	Mycoplasmataceae
Proteobacteria	Betaproteobacteria	Burkholderiales	Oxalobacteraceae
Proteobacteria	Gammaproteobacteria	Pasteurellales	Pasteurellaceae
Planctomycetes	Planctomycetia	Planctomycetales	Planctomycetaceae
Firmicutes	Bacilli	Bacillales	Planococcaceae
Bacteroidetes	Bacteroidia	Bacteroidales	Porphyromonadaceae
Bacteroidetes	Bacteroidia	Bacteroidales	Prevotellaceae

Bacteroidetes	Bacteroidia	Bacteroidales	Prolixibacteraceae
Proteobacteria	Alphaproteobacteria	Rhodobacterales	Rhodobacteraceae
Proteobacteria	Alphaproteobacteria	Rhodospirillales	Rhodospirillaceae
Bacteroidetes	Bacteroidia	Bacteroidales	Rikenellaceae
Firmicutes	Clostridia	Clostridiales	Ruminococcaceae
Spirochaetes	Spirochaetia	Spirochaetales	Spirochaetaceae
Proteobacteria	Betaproteobacteria	Burkholderiales	Sutterellaceae
Synergistetes	Synergistia	Synergistales	Synergistaceae
Firmicutes	Negativicutes	Selenomonadales	Veillonellaceae
