# Supplementary material for: The equine gastrointestinal microbiome: impacts of weight-loss
Source: BMC Vet Res. 2020 Mar 4;16:78. doi: 10.1186/s12917-020-02295-6 (PMC7057583; doi:10.1186/s12917-020-02295-6)
Supplement: Supplementary file 10 — Additional File 10. Relative abundance of outset bacterial genera (mean of 3 pre-diet days) between the three weight-loss groups (n = 5/group). [file 12917_2020_2295_MOESM10_ESM.pdf]

Additional File 10. Relative abundance of outset bacterial genera (mean of 3 pre-diet days) between the three weight-loss groups (n = 5/group).

	**Low**	**Mid**	**High**	**SED**	**Benjamini-Hochberg P-value**
Unclassified	0.691	0.616	0.581	0.032	0.434
Fibrobacter	0.083	0.181	0.221	0.070	0.328
Treponema	0.034	0.034	0.016	0.020	0.205
Phascolarctobacterium	0.026	0.018	0.013	0.019	0.424
Alkalitalea	0.023	0.016	0.021	0.069	0.880
Prevotella	0.015	0.013	0.020	0.029	0.872
Paraprevotella	0.013	0.015	0.012	0.020	0.880
Barnesiella	0.011	0.004	0.026	0.043	0.690
Ruminococcus	0.011	0.012	0.009	0.018	0.872
Paludibacter	0.010	0.015	0.022	0.041	0.872
Alloprevotella	0.009	0.004	0.002	0.023	0.706
Clostridium XIVa	0.008	0.005	0.005	0.010	0.476
Oscillibacter	0.008	0.007	0.006	0.010	0.872
Phocaeicola	0.006	0.011	0.009	0.026	0.872
Anaeroplasma	0.006	0.003	0.003	0.012	0.559
Rikenella	0.006	0.009	0.006	0.010	0.434
Lachnospiracea	0.006	0.005	0.004	0.007	0.770
Faecalitalea	0.003	0.002	0.001	0.010	0.434
Asteroleplasma	0.003	0.001	0.001	0.016	0.872
Anaerovorax	0.002	0.002	0.001	0.005	0.144
Coprobacter	0.002	0.002	0.001	0.012	0.750
Sporobacter	0.002	0.002	0.001	0.004	0.328
Sphaerochaeta	0.002	0.001	0.000	0.007	0.150
Macellibacteroides	0.002	0.001	0.000	0.019	0.872
Pseudoflavonifractor	0.002	0.009	0.004	0.030	0.872
Lachnobacterium	0.002	0.001	0.001	0.008	0.872
Intestinimonas	0.002	0.002	0.003	0.007	0.640
Clostridium IV	0.002	0.002	0.002	0.005	0.706
Faecalicoccus	0.001	0.001	0.000	0.006	0.328
Mobilitalea	0.001	0.001	0.000	0.004	0.041
Anaerorhabdus	0.001	0.000	0.001	0.009	0.746
Roseburia	0.001	0.001	0.001	0.004	0.872
Saccharofermentans	0.001	0.000	0.001	0.005	0.690
Vampirovibrio	0.001	0.001	0.001	0.005	0.872
Saccharibacteria	0.001	0.001	0.001	0.005	0.872
Ethanoligenens	0.001	0.001	0.001	0.008	0.872
Anaerocella	0.001	0.001	0.000	0.006	0.872
Mogibacterium	0.001	0.001	0.001	0.005	0.872
Candidatusendomicrobium	0.000	0.000	0.001	0.007	0.915
Catabacter	0.000	0.000	0.000	0.004	0.872
Streptococcus	0.000	0.000	0.001	0.007	0.287

ANOVA analysis was employed to evaluate group differences in the relative abundance of bacterial phyla, and the resulting P-values were adjusted for multiple testing using the Benjamini-Hochberg correction.
