# Supplementary material for: The equine gastrointestinal microbiome: impacts of weight-loss
Source: BMC Vet Res. 2020 Mar 4;16:78. doi: 10.1186/s12917-020-02295-6 (PMC7057583; doi:10.1186/s12917-020-02295-6)
Supplement: Supplementary file 11 — Additional File 11. Relative abundance of bacterial OTUs significantly different in abundance before (pre-diet), and after 7 weeks of dietary restriction (post-diet; n = 15). [file 12917_2020_2295_MOESM11_ESM.pdf]

Additional File 11. Relative abundance of bacterial OTUs significantly different in abundance before (pre-diet), and after 7 weeks of dietary restriction (post-diet; n = 15).

Phylum	Class	Order	Family	Genus	log2FoldChange	Adjusted P-value	Relative abundance	
							Pre-diet	Post-diet
Unclassified	*Unclassified*	*Unclassified*	*Unclassified*	*Unclassified*	-6.638	0.000	0.102	0.217
Firmicutes	*Clostridia*	*Clostridiales*	*Ruminococcaceae*	*Ruminococcus*	-5.062	0.002	0.020	0.169
Bacteroidetes	*Bacteroidia*	*Bacteroidales*	*Unclassified*	*Unclassified*	-4.967	0.054	0.050	0.104
Firmicutes	*Clostridia*	*Clostridiales*	*Lachnospiraceae*	*Unclassified*	-4.619	0.002	0.004	0.056
Bacteroidetes	*Bacteroidia*	*Bacteroidales*	*Unclassified*	*Unclassified*	-4.545	0.003	0.141	1.797
Bacteroidetes	*Bacteroidia*	*Bacteroidales*	*Unclassified*	*Unclassified*	-4.284	0.038	0.043	0.143
Spirochaetes	*Spirochaetia*	*Spirochaetales*	*Spirochaetaceae*	*Treponema*	-4.214	0.037	0.033	0.084
Spirochaetes	*Spirochaetia*	*Spirochaetales*	*Spirochaetaceae*	*Treponema*	-3.961	0.005	0.026	0.084
Firmicutes	*Erysipelotrichia*	*Erysipelotrichales*	*Erysipelotrichaceae*	*Unclassified*	-3.882	0.002	0.001	0.039
Bacteroidetes	*Unclassified*	*Unclassified*	*Unclassified*	*Unclassified*	-3.764	0.016	0.017	0.088
Spirochaetes	*Spirochaetia*	*Spirochaetales*	*Spirochaetaceae*	*Unclassified*	-3.713	0.002	0.005	0.096
Bacteroidetes	*Unclassified*	*Unclassified*	*Unclassified*	*Unclassified*	-3.496	0.002	0.101	0.741
Spirochaetes	*Spirochaetia*	*Spirochaetales*	*Spirochaetaceae*	*Treponema*	-3.232	0.022	0.268	0.574
Unclassified	*Unclassified*	*Unclassified*	*Unclassified*	*Unclassified*	-3.135	0.041	0.034	0.066
Firmicutes	*Clostridia*	*Clostridiales*	*Lachnospiraceae*	*Unclassified*	-2.836	0.088	0.005	0.031
Firmicutes	*Clostridia*	*Clostridiales*	*Lachnospiraceae*	*Unclassified*	-2.818	0.098	0.022	0.043
Bacteroidetes	*Bacteroidia*	*Bacteroidales*	*Unclassified*	*Unclassified*	-2.816	0.005	0.030	0.055
Firmicutes	*Clostridia*	*Clostridiales*	*Lachnospiraceae*	*Unclassified*	-2.728	0.037	0.011	0.035
Firmicutes	*Unclassified*	*Unclassified*	*Unclassified*	*Unclassified*	-2.575	0.052	0.008	0.020
Bacteroidetes	*Bacteroidia*	*Bacteroidales*	*Rikenellaceae*	*Unclassified*	-2.575	0.091	0.024	0.008

Spirochaetes	*Spirochaetia*	*Spirochaetales*	*Spirochaetaceae*	*Treponema*	-2.553	0.054	0.076	0.127
Bacteroidetes	*Bacteroidia*	*Bacteroidales*	*Unclassified*	*Unclassified*	-2.291	0.069	0.224	0.576
Bacteroidetes	*Bacteroidia*	*Bacteroidales*	*Unclassified*	*Unclassified*	-2.253	0.038	0.035	0.057
Bacteroidetes	*Unclassified*	*Unclassified*	*Unclassified*	*Unclassified*	-2.217	0.088	0.017	0.244
Spirochaetes	*Spirochaetia*	*Spirochaetales*	*Spirochaetaceae*	*Treponema*	-2.217	0.048	0.060	0.133
Fibrobacteres	*Fibrobacteria*	*Fibrobacterales*	*Fibrobacteraceae*	*Fibrobacter*	-2.209	0.097	10.331	17.837
Fibrobacteres	*Fibrobacteria*	*Fibrobacterales*	*Fibrobacteraceae*	*Fibrobacter*	-2.178	0.085	0.035	0.020
Bacteroidetes	*Unclassified*	*Unclassified*	*Unclassified*	*Unclassified*	-2.176	0.097	0.339	0.547
Bacteroidetes	*Bacteroidia*	*Bacteroidales*	*Rikenellaceae*	*Rikenella*	-2.159	0.085	0.018	0.025
Bacteroidetes	*Bacteroidia*	*Bacteroidales*	*Rikenellaceae*	*Rikenella*	-2.127	0.048	0.098	0.170
Bacteroidetes	*Bacteroidia*	*Bacteroidales*	*Unclassified*	*Unclassified*	-2.127	0.037	0.017	0.044
Unclassified	*Unclassified*	*Unclassified*	*Unclassified*	*Unclassified*	-2.112	0.096	0.017	0.050
Bacteroidetes	*Bacteroidia*	*Bacteroidales*	*Bacteroidaceae*	*Anaerorhabdus*	-2.092	0.005	0.177	0.489
Bacteroidetes	*Unclassified*	*Unclassified*	*Unclassified*	*Unclassified*	-2.075	0.085	0.024	0.011
Firmicutes	*Clostridia*	*Clostridiales*	*Catabacteriaceae*	*Catabacter*	-2.020	0.023	0.007	0.032
Firmicutes	*Clostridia*	*Clostridiales*	*Ruminococcaceae*	*Unclassified*	-2.019	0.097	0.068	0.207
Firmicutes	*Negativicutes*	*Selenomonadales*	*Acidaminococcaceae*	*Phascolarctobacterium*	-1.969	0.079	0.014	0.043
Firmicutes	*Clostridia*	*Clostridiales*	*Ruminococcaceae*	*Intestinimonas*	-1.892	0.086	0.007	0.023
Firmicutes	*Clostridia*	*Clostridiales*	*Unclassified*	*Unclassified*	-1.818	0.062	0.013	0.022
Bacteroidetes	*Bacteroidia*	*Bacteroidales*	*Unclassified*	*Unclassified*	-1.770	0.097	0.006	0.021
Bacteroidetes	*Unclassified*	*Unclassified*	*Unclassified*	*Unclassified*	-1.707	0.085	0.076	0.117
Bacteroidetes	*Bacteroidia*	*Bacteroidales*	*Unclassified*	*Unclassified*	-1.687	0.037	0.039	0.102
Bacteroidetes	*Bacteroidia*	*Bacteroidales*	*Unclassified*	*Unclassified*	-1.636	0.085	0.031	0.096
Unclassified	*Unclassified*	*Unclassified*	*Unclassified*	*Unclassified*	-1.602	0.037	0.002	0.160
Firmicutes	*Clostridia*	*Clostridiales*	*Unclassified*	*Unclassified*	-1.565	0.093	0.014	0.032
Bacteroidetes	*Bacteroidia*	*Bacteroidales*	*Unclassified*	*Unclassified*	-1.539	0.098	0.008	0.017

Firmicutes	*Clostridia*	*Clostridiales*	*Unclassified*	*Unclassified*	-1.505	0.088	0.039	0.098
Bacteroidetes	*Bacteroidia*	*Bacteroidales*	*Unclassified*	*Unclassified*	-1.293	0.022	0.010	0.027
Firmicutes	*Clostridia*	*Clostridiales*	*Ruminococcaceae*	*Unclassified*	-1.292	0.079	0.009	0.017
Firmicutes	*Erysipelotrichia*	*Erysipelotrichales*	*Erysipelotrichaceae*	*Holdemania*	-1.201	0.085	0.008	0.016
Firmicutes	*Clostridia*	*Clostridiales*	*Ruminococcaceae*	*Unclassified*	-1.198	0.048	0.049	0.124
Firmicutes	*Clostridia*	*Clostridiales*	*Unclassified*	*Unclassified*	-1.157	0.079	0.009	0.022
Firmicutes	*Clostridia*	*Clostridiales*	*Unclassified*	*Unclassified*	-1.100	0.097	0.019	0.039
Firmicutes	*Clostridia*	*Clostridiales*	*Ruminococcaceae*	*Anaerobacterium*	-0.800	0.097	0.024	0.041
Firmicutes	*Clostridia*	*Clostridiales*	*Ruminococcaceae*	*Oscillibacter*	0.644	0.097	0.048	0.025
Firmicutes	*Clostridia*	*Clostridiales*	*Lachnospiraceae*	*Unclassified*	0.879	0.085	0.084	0.126
Firmicutes	*Clostridia*	*Clostridiales*	*Lachnospiraceae*	*Lachnospiracea_incertae_sedis*	0.884	0.054	0.041	0.020
Firmicutes	*Clostridia*	*Clostridiales*	*Unclassified*	*Unclassified*	1.288	0.097	0.038	0.008
Firmicutes	*Clostridia*	*Clostridiales*	*Lachnospiraceae*	*Unclassified*	1.569	0.091	1.694	0.450
Bacteroidetes	*Bacteroidia*	*Bacteroidales*	*Unclassified*	*Unclassified*	1.960	0.044	0.057	0.009
Firmicutes	*Clostridia*	*Clostridiales*	*Ruminococcaceae*	*Unclassified*	2.518	0.014	0.529	0.265
