# Supplementary material for: The equine gastrointestinal microbiome: impacts of weight-loss
Source: BMC Vet Res. 2020 Mar 4;16:78. doi: 10.1186/s12917-020-02295-6 (PMC7057583; doi:10.1186/s12917-020-02295-6)
Supplement: Supplementary file 12 — Additional File 12. Relative abundance of outset bacterial OTUs significantly different in abundance between high and low weight-loss groups (n = 5/group). [file 12917_2020_2295_MOESM12_ESM.pdf]

Additional File 12. Relative abundance of outset bacterial OTUs significantly different in abundance between high and low weight-loss groups (n = 5/group).

Phylum	Class	Order	Family	Genus	log2Fold Change	Adjusted P-value	Relative abundance	
							Low	High
Firmicutes	*Negativicutes*	*Selenomonadales*	*Acidaminococcaceae*	*Phascolarctobacterium*	2.983	0.036	0.074	0.004
Firmicutes	*Negativicutes*	*Selenomonadales*	*Acidaminococcaceae*	*Phascolarctobacterium*	3.350	0.038	0.036	0.002
Bacteroidetes	*Bacteroidia*	*Bacteroidales*	*Bacteroidales_incertae_sedis*	*Phocaeicola*	-23.961	0.000	0.000	0.087
Bacteroidetes	*Bacteroidia*	*Bacteroidales*	*Bacteroidales_incertae_sedis*	*Phocaeicola*	-3.140	0.015	0.006	0.022
Bacteroidetes	*Bacteroidia*	*Bacteroidales*	*Bacteroidales_incertae_sedis*	*Phocaeicola*	-8.973	0.020	0.000	0.083
Proteobacteria	*Epsilonproteobacteria*	*Campylobacterales*	*Campylobacteraceae*	*Campylobacter*	2.957	0.061	0.035	0.002
Actinobacteria	*Actinobacteria*	*Coriobacteriales*	*Coriobacteriaceae*	*Unclassified*	1.906	0.051	0.025	0.003
Firmicutes	*Erysipelotrichia*	*Erysipelotrichales*	*Erysipelotrichaceae*	*Faecalitalea*	2.643	0.054	0.025	0.002
Firmicutes	*Erysipelotrichia*	*Erysipelotrichales*	*Erysipelotrichaceae*	*Faecalitalea*	2.129	0.058	0.133	0.013
Fibrobacteres	*Fibrobacteria*	*Fibrobacterales*	*Fibrobacteraceae*	*Fibrobacter*	-10.243	0.000	0.008	3.536
Fibrobacteres	*Fibrobacteria*	*Fibrobacterales*	*Fibrobacteraceae*	*Fibrobacter*	-9.252	0.000	0.000	0.131
Fibrobacteres	*Fibrobacteria*	*Fibrobacterales*	*Fibrobacteraceae*	*Fibrobacter*	-9.470	0.000	0.000	0.102
Fibrobacteres	*Fibrobacteria*	*Fibrobacterales*	*Fibrobacteraceae*	*Fibrobacter*	-7.300	0.002	0.000	0.022
Fibrobacteres	*Fibrobacteria*	*Fibrobacterales*	*Fibrobacteraceae*	*Fibrobacter*	-11.246	0.003	0.001	1.013
Fibrobacteres	*Fibrobacteria*	*Fibrobacterales*	*Fibrobacteraceae*	*Fibrobacter*	-7.500	0.010	0.000	0.025
Fibrobacteres	*Fibrobacteria*	*Fibrobacterales*	*Fibrobacteraceae*	*Fibrobacter*	-3.297	0.020	0.030	0.185
Fibrobacteres	*Fibrobacteria*	*Fibrobacterales*	*Fibrobacteraceae*	*Fibrobacter*	-4.198	0.033	0.004	0.025
Fibrobacteres	*Fibrobacteria*	*Fibrobacterales*	*Fibrobacteraceae*	*Fibrobacter*	-4.064	0.057	0.010	0.055
Fibrobacteres	*Fibrobacteria*	*Fibrobacterales*	*Fibrobacteraceae*	*Fibrobacter*	-3.872	0.061	0.109	0.788

Fibrobacteres	*Fibrobacteria*	*Fibrobacterales*	*Fibrobacteraceae*	*Fibrobacter*	-4.585	0.061	0.038	0.288
Fibrobacteres	*Fibrobacteria*	*Fibrobacterales*	*Fibrobacteraceae*	*Fibrobacter*	-4.213	0.078	0.048	0.280
Fibrobacteres	*Fibrobacteria*	*Fibrobacterales*	*Fibrobacteraceae*	*Fibrobacter*	-5.866	0.081	0.001	0.016
Firmicutes	*Clostridia*	*Clostridiales*	*Lachnospiraceae*	*Lachnospiracea_incertae_sedis*	-1.829	0.039	0.027	0.039
Firmicutes	*Clostridia*	*Clostridiales*	*Lachnospiraceae*	*Lachnospiracea_incertae_sedis*	-1.950	0.049	0.008	0.013
Firmicutes	*Clostridia*	*Clostridiales*	*Lachnospiraceae*	*Lachnospiracea_incertae_sedis*	-4.620	0.061	0.003	0.057
Firmicutes	*Clostridia*	*Clostridiales*	*Lachnospiraceae*	*Lachnospiracea_incertae_sedis*	-1.240	0.093	0.014	0.015
Firmicutes	*Clostridia*	*Clostridiales*	*Lachnospiraceae*	*Mobilitalea*	2.846	0.006	0.084	0.006
Firmicutes	*Clostridia*	*Clostridiales*	*Lachnospiraceae*	*Unclassified*	-7.016	0.000	0.003	0.172
Firmicutes	*Clostridia*	*Clostridiales*	*Lachnospiraceae*	*Unclassified*	-5.897	0.000	0.003	0.066
Firmicutes	*Clostridia*	*Clostridiales*	*Lachnospiraceae*	*Unclassified*	-3.069	0.007	0.009	0.041
Firmicutes	*Clostridia*	*Clostridiales*	*Lachnospiraceae*	*Unclassified*	-2.709	0.008	0.018	0.054
Firmicutes	*Clostridia*	*Clostridiales*	*Lachnospiraceae*	*Unclassified*	-4.754	0.025	0.002	0.020
Firmicutes	*Clostridia*	*Clostridiales*	*Lachnospiraceae*	*Unclassified*	-1.769	0.025	0.010	0.016
Firmicutes	*Clostridia*	*Clostridiales*	*Lachnospiraceae*	*Unclassified*	-1.625	0.094	0.053	0.093
Bacteroidetes	*Bacteroidia*	*Bacteroidales*	*Marinilabiliaceae*	*Alkalitalea*	-27.694	0.000	0.000	1.431
Bacteroidetes	*Bacteroidia*	*Bacteroidales*	*Porphyromonadaceae*	*Barnesiella*	-3.783	0.026	0.660	2.519
Bacteroidetes	*Bacteroidia*	*Bacteroidales*	*Porphyromonadaceae*	*Paludibacter*	-5.823	0.006	0.002	0.065
Bacteroidetes	*Bacteroidia*	*Bacteroidales*	*Porphyromonadaceae*	*Paludibacter*	-4.363	0.081	0.110	0.762
Bacteroidetes	*Bacteroidia*	*Bacteroidales*	*Porphyromonadaceae*	*Unclassified*	20.356	0.000	0.070	0.000
Bacteroidetes	*Bacteroidia*	*Bacteroidales*	*Porphyromonadaceae*	*Unclassified*	-25.588	0.000	0.000	0.283
Bacteroidetes	*Bacteroidia*	*Bacteroidales*	*Porphyromonadaceae*	*Unclassified*	13.190	0.002	0.000	0.000

Bacteroidetes	*Bacteroidia*	*Bacteroidales*	*Porphyromonadaceae*	*Unclassified*	2.806	0.003	0.061	0.005
Bacteroidetes	*Bacteroidia*	*Bacteroidales*	*Porphyromonadaceae*	*Unclassified*	6.084	0.004	0.060	0.000
Bacteroidetes	*Bacteroidia*	*Bacteroidales*	*Porphyromonadaceae*	*Unclassified*	-7.846	0.016	0.000	0.035
Bacteroidetes	*Bacteroidia*	*Bacteroidales*	*Porphyromonadaceae*	*Unclassified*	5.697	0.025	0.030	0.000
Bacteroidetes	*Bacteroidia*	*Bacteroidales*	*Porphyromonadaceae*	*Unclassified*	-3.111	0.049	0.137	0.475
Bacteroidetes	*Bacteroidia*	*Bacteroidales*	*Porphyromonadaceae*	*Unclassified*	-3.587	0.049	0.005	0.024
Bacteroidetes	*Bacteroidia*	*Bacteroidales*	*Porphyromonadaceae*	*Unclassified*	3.679	0.057	0.976	0.034
Bacteroidetes	*Bacteroidia*	*Bacteroidales*	*Porphyromonadaceae*	*Unclassified*	-3.663	0.081	0.175	1.552
Bacteroidetes	*Bacteroidia*	*Bacteroidales*	*Prevotellaceae*	*Paraprevotella*	1.356	0.057	0.065	0.012
Bacteroidetes	*Bacteroidia*	*Bacteroidales*	*Prevotellaceae*	*Paraprevotella*	2.791	0.057	0.161	0.014
Bacteroidetes	*Bacteroidia*	*Bacteroidales*	*Prevotellaceae*	*Paraprevotella*	1.380	0.093	0.141	0.025
Bacteroidetes	*Bacteroidia*	*Bacteroidales*	*Prevotellaceae*	*Prevotella*	-4.966	0.001	0.041	0.645
Bacteroidetes	*Bacteroidia*	*Bacteroidales*	*Prevotellaceae*	*Prevotella*	-4.349	0.011	0.003	0.030
Bacteroidetes	*Bacteroidia*	*Bacteroidales*	*Prevotellaceae*	*Unclassified*	-28.067	0.000	0.000	2.273
Bacteroidetes	*Bacteroidia*	*Bacteroidales*	*Prevotellaceae*	*Unclassified*	-26.402	0.000	0.000	0.736
Bacteroidetes	*Bacteroidia*	*Bacteroidales*	*Prevotellaceae*	*Unclassified*	1.811	0.072	0.103	0.013
Bacteroidetes	*Bacteroidia*	*Bacteroidales*	*Rikenellaceae*	*Rikenella*	-5.849	0.093	0.006	0.164
Bacteroidetes	*Bacteroidia*	*Bacteroidales*	*Rikenellaceae*	*Rikenella*	-4.225	0.094	0.002	0.016
Bacteroidetes	*Bacteroidia*	*Bacteroidales*	*Rikenellaceae*	*Unclassified*	-3.008	0.004	0.028	0.103
Firmicutes	*Clostridia*	*Clostridiales*	*Ruminococcaceae*	*Oscillibacter*	-3.781	0.020	0.009	0.041
Firmicutes	*Clostridia*	*Clostridiales*	*Ruminococcaceae*	*Oscillibacter*	-2.218	0.061	0.010	0.017
Firmicutes	*Clostridia*	*Clostridiales*	*Ruminococcaceae*	*Oscillibacter*	2.535	0.061	0.125	0.010
Firmicutes	*Clostridia*	*Clostridiales*	*Ruminococcaceae*	*Oscillibacter*	-1.584	0.081	0.024	0.027
Firmicutes	*Clostridia*	*Clostridiales*	*Ruminococcaceae*	*Oscillibacter*	-2.376	0.099	0.010	0.022
Firmicutes	*Clostridia*	*Clostridiales*	*Ruminococcaceae*	*Ruminococcus*	-8.090	0.006	0.000	0.050
Firmicutes	*Clostridia*	*Clostridiales*	*Ruminococcaceae*	*Ruminococcus*	-4.211	0.061	0.004	0.034

Firmicutes	*Clostridia*	*Clostridiales*	*Ruminococcaceae*	*Unclassified*	-5.279	0.001	0.116	1.364
Firmicutes	*Clostridia*	*Clostridiales*	*Ruminococcaceae*	*Unclassified*	3.750	0.011	0.336	0.013
Firmicutes	*Clostridia*	*Clostridiales*	*Ruminococcaceae*	*Unclassified*	2.894	0.038	0.036	0.003
Spirochaetes	*Spirochaetia*	*Spirochaetales*	*Spirochaetaceae*	*Sphaerochaeta*	3.309	0.002	0.131	0.007
Spirochaetes	*Spirochaetia*	*Spirochaetales*	*Spirochaetaceae*	*Treponema*	-6.006	0.001	0.003	0.084
Spirochaetes	*Spirochaetia*	*Spirochaetales*	*Spirochaetaceae*	*Treponema*	-7.024	0.008	0.000	0.024
Spirochaetes	*Spirochaetia*	*Spirochaetales*	*Spirochaetaceae*	*Treponema*	-8.193	0.010	0.001	0.081
Spirochaetes	*Spirochaetia*	*Spirochaetales*	*Spirochaetaceae*	*Treponema*	6.873	0.016	0.100	0.000
Spirochaetes	*Spirochaetia*	*Spirochaetales*	*Spirochaetaceae*	*Treponema*	-6.341	0.049	0.002	0.056
Spirochaetes	*Spirochaetia*	*Spirochaetales*	*Spirochaetaceae*	*Treponema*	6.970	0.093	0.074	0.000
Bacteroidetes	*Bacteroidia*	*Bacteroidales*	*Unclassified*	*Unclassified*	-23.485	0.000	0.000	0.049
Bacteroidetes	*Bacteroidia*	*Bacteroidales*	*Unclassified*	*Unclassified*	-23.648	0.000	0.000	0.062
Bacteroidetes	*Bacteroidia*	*Bacteroidales*	*Unclassified*	*Unclassified*	-24.272	0.000	0.000	0.112
Bacteroidetes	*Bacteroidia*	*Bacteroidales*	*Unclassified*	*Unclassified*	24.815	0.000	1.527	0.000
Bacteroidetes	*Unclassified*	*Unclassified*	*Unclassified*	*Unclassified*	-23.955	0.000	0.000	0.070
Bacteroidetes	*Bacteroidia*	*Bacteroidales*	*Unclassified*	*Unclassified*	-22.202	0.000	0.000	0.022
Bacteroidetes	*Bacteroidia*	*Bacteroidales*	*Unclassified*	*Unclassified*	-9.089	0.000	0.000	0.107
Bacteroidetes	*Unclassified*	*Unclassified*	*Unclassified*	*Unclassified*	-7.483	0.001	0.001	0.068
Bacteroidetes	*Bacteroidia*	*Bacteroidales*	*Unclassified*	*Unclassified*	-6.012	0.001	0.002	0.041
Bacteroidetes	*Unclassified*	*Unclassified*	*Unclassified*	*Unclassified*	3.215	0.003	0.036	0.002
Bacteroidetes	*Bacteroidia*	*Bacteroidales*	*Unclassified*	*Unclassified*	4.978	0.003	0.026	0.000
Bacteroidetes	*Bacteroidia*	*Bacteroidales*	*Unclassified*	*Unclassified*	-5.048	0.004	0.002	0.029
Bacteroidetes	*Unclassified*	*Unclassified*	*Unclassified*	*Unclassified*	-4.000	0.005	0.009	0.046
Bacteroidetes	*Bacteroidia*	*Bacteroidales*	*Unclassified*	*Unclassified*	4.492	0.006	0.115	0.003
Bacteroidetes	*Bacteroidia*	*Bacteroidales*	*Unclassified*	*Unclassified*	-4.896	0.007	0.009	0.204
Bacteroidetes	*Bacteroidia*	*Bacteroidales*	*Unclassified*	*Unclassified*	-4.764	0.007	0.007	0.079

Unclassified	*Unclassified*	*Unclassified*	*Unclassified*	*Unclassified*	2.906	0.009	0.431	0.024
Firmicutes	*Clostridia*	*Clostridiales*	*Unclassified*	*Unclassified*	3.826	0.013	0.042	0.001
Bacteroidetes	*Bacteroidia*	*Bacteroidales*	*Unclassified*	*Unclassified*	3.716	0.014	0.052	0.002
Bacteroidetes	*Unclassified*	*Unclassified*	*Unclassified*	*Unclassified*	3.995	0.015	0.039	0.001
Bacteroidetes	*Unclassified*	*Unclassified*	*Unclassified*	*Unclassified*	-4.562	0.016	0.063	0.534
Firmicutes	*Clostridia*	*Clostridiales*	*Unclassified*	*Unclassified*	-7.699	0.018	0.000	0.029
Unclassified	*Unclassified*	*Unclassified*	*Unclassified*	*Unclassified*	2.848	0.020	0.030	0.002
Firmicutes	*Unclassified*	*Unclassified*	*Unclassified*	*Unclassified*	3.794	0.022	0.067	0.002
Bacteroidetes	*Bacteroidia*	*Bacteroidales*	*Unclassified*	*Unclassified*	2.727	0.023	0.066	0.005
Bacteroidetes	*Unclassified*	*Unclassified*	*Unclassified*	*Unclassified*	-5.858	0.024	0.001	0.050
Bacteroidetes	*Bacteroidia*	*Bacteroidales*	*Unclassified*	*Unclassified*	2.088	0.024	0.047	0.005
Bacteroidetes	*Unclassified*	*Unclassified*	*Unclassified*	*Unclassified*	-4.138	0.025	0.009	0.080
Bacteroidetes	*Bacteroidia*	*Bacteroidales*	*Unclassified*	*Unclassified*	-5.644	0.026	0.001	0.024
Bacteroidetes	*Unclassified*	*Unclassified*	*Unclassified*	*Unclassified*	2.435	0.026	0.054	0.005
Bacteroidetes	*Bacteroidia*	*Bacteroidales*	*Unclassified*	*Unclassified*	-8.066	0.026	0.000	0.051
Bacteroidetes	*Unclassified*	*Unclassified*	*Unclassified*	*Unclassified*	-2.276	0.028	0.013	0.029
Proteobacteria	*Alphaproteobacteria*	*Unclassified*	*Unclassified*	*Unclassified*	4.941	0.028	0.025	0.000
Bacteroidetes	*Unclassified*	*Unclassified*	*Unclassified*	*Unclassified*	2.860	0.028	0.054	0.004
Bacteroidetes	*Bacteroidia*	*Bacteroidales*	*Unclassified*	*Unclassified*	4.682	0.028	0.122	0.002
Bacteroidetes	*Bacteroidia*	*Bacteroidales*	*Unclassified*	*Unclassified*	-5.445	0.028	0.002	0.026
Bacteroidetes	*Bacteroidia*	*Bacteroidales*	*Unclassified*	*Unclassified*	-7.501	0.030	0.000	0.032
Unclassified	*Unclassified*	*Unclassified*	*Unclassified*	*Unclassified*	2.076	0.033	0.041	0.005
Bacteroidetes	*Bacteroidia*	*Bacteroidales*	*Unclassified*	*Unclassified*	4.789	0.034	0.070	0.001
Bacteroidetes	*Bacteroidia*	*Bacteroidales*	*Unclassified*	*Unclassified*	2.383	0.036	0.047	0.004
Bacteroidetes	*Bacteroidia*	*Bacteroidales*	*Unclassified*	*Unclassified*	-7.228	0.036	0.001	0.073
Unclassified	*Unclassified*	*Unclassified*	*Unclassified*	*Unclassified*	2.513	0.038	0.084	0.007

Bacteroidetes	*Unclassified*	*Unclassified*	*Unclassified*	*Unclassified*	-3.208	0.038	0.007	0.033
Bacteroidetes	*Bacteroidia*	*Bacteroidales*	*Unclassified*	*Unclassified*	4.044	0.041	0.042	0.001
Bacteroidetes	*Bacteroidia*	*Bacteroidales*	*Unclassified*	*Unclassified*	-8.993	0.045	0.000	0.071
Bacteroidetes	*Unclassified*	*Unclassified*	*Unclassified*	*Unclassified*	-4.319	0.048	0.007	0.041
Bacteroidetes	*Unclassified*	*Unclassified*	*Unclassified*	*Unclassified*	-7.526	0.049	0.000	0.026
Firmicutes	*Unclassified*	*Unclassified*	*Unclassified*	*Unclassified*	1.769	0.049	0.036	0.006
Bacteroidetes	*Unclassified*	*Unclassified*	*Unclassified*	*Unclassified*	-4.597	0.051	0.023	0.188
Firmicutes	*Clostridia*	*Clostridiales*	*Unclassified*	*Unclassified*	1.887	0.054	0.028	0.004
Proteobacteria	*Betaproteobacteria*	*Unclassified*	*Unclassified*	*Unclassified*	2.389	0.054	0.054	0.005
Bacteroidetes	*Unclassified*	*Unclassified*	*Unclassified*	*Unclassified*	-8.608	0.058	0.001	0.112
Firmicutes	*Clostridia*	*Clostridiales*	*Unclassified*	*Unclassified*	-2.236	0.058	0.007	0.013
Bacteroidetes	*Bacteroidia*	*Bacteroidales*	*Unclassified*	*Unclassified*	2.144	0.058	0.298	0.035
Bacteroidetes	*Cytophagia*	*Cytophagales*	*Unclassified*	*Unclassified*	-6.078	0.058	0.001	0.030
Bacteroidetes	*Bacteroidia*	*Bacteroidales*	*Unclassified*	*Unclassified*	-4.014	0.059	0.011	0.077
Bacteroidetes	*Unclassified*	*Unclassified*	*Unclassified*	*Unclassified*	-3.174	0.061	0.008	0.031
Bacteroidetes	*Bacteroidia*	*Bacteroidales*	*Unclassified*	*Unclassified*	-3.365	0.061	0.006	0.026
Bacteroidetes	*Bacteroidia*	*Bacteroidales*	*Unclassified*	*Unclassified*	-4.297	0.061	0.000	0.005
Proteobacteria	*Unclassified*	*Unclassified*	*Unclassified*	*Unclassified*	5.961	0.061	0.081	0.001
Firmicutes	*Unclassified*	*Unclassified*	*Unclassified*	*Unclassified*	1.733	0.061	0.032	0.004
Bacteroidetes	*Bacteroidia*	*Bacteroidales*	*Unclassified*	*Unclassified*	-2.148	0.061	0.021	0.050
Unclassified	*Unclassified*	*Unclassified*	*Unclassified*	*Unclassified*	-1.896	0.061	0.053	0.085
Bacteroidetes	*Bacteroidia*	*Bacteroidales*	*Unclassified*	*Unclassified*	2.105	0.061	0.043	0.005
Bacteroidetes	*Unclassified*	*Unclassified*	*Unclassified*	*Unclassified*	1.861	0.061	0.043	0.006
Firmicutes	*Clostridia*	*Clostridiales*	*Unclassified*	*Unclassified*	-2.846	0.061	0.003	0.011
Bacteroidetes	*Unclassified*	*Unclassified*	*Unclassified*	*Unclassified*	-4.725	0.062	0.004	0.031
Unclassified	*Unclassified*	*Unclassified*	*Unclassified*	*Unclassified*	-1.797	0.065	0.010	0.015

Bacteroidetes	*Unclassified*	*Unclassified*	*Unclassified*	*Unclassified*	4.968	0.069	0.115	0.001
Firmicutes	*Clostridia*	*Clostridiales*	*Unclassified*	*Unclassified*	3.469	0.074	0.036	0.001
Bacteroidetes	*Bacteroidia*	*Bacteroidales*	*Unclassified*	*Unclassified*	-3.199	0.076	0.005	0.019
Bacteroidetes	*Bacteroidia*	*Bacteroidales*	*Unclassified*	*Unclassified*	-8.376	0.076	0.000	0.040
Bacteroidetes	*Bacteroidia*	*Bacteroidales*	*Unclassified*	*Unclassified*	2.965	0.076	0.456	0.030
Bacteroidetes	*Unclassified*	*Unclassified*	*Unclassified*	*Unclassified*	-1.930	0.085	0.012	0.019
Unclassified	*Unclassified*	*Unclassified*	*Unclassified*	*Unclassified*	-5.400	0.085	0.001	0.013
Bacteroidetes	*Cytophagia*	*Cytophagales*	*Unclassified*	*Unclassified*	-3.720	0.085	0.055	0.379
Firmicutes	*Clostridia*	*Clostridiales*	*Unclassified*	*Unclassified*	2.826	0.089	0.054	0.003
Firmicutes	*Unclassified*	*Unclassified*	*Unclassified*	*Unclassified*	-3.231	0.090	0.016	0.055
Firmicutes	*Clostridia*	*Clostridiales*	*Unclassified*	*Unclassified*	-2.108	0.093	0.006	0.011
Bacteroidetes	*Bacteroidia*	*Bacteroidales*	*Unclassified*	*Unclassified*	-2.211	0.096	0.010	0.020
Unclassified	*Unclassified*	*Unclassified*	*Unclassified*	*Unclassified*	2.511	0.097	0.058	0.005
Firmicutes	*Negativicutes*	*Selenomonadales*	*Veillonellaceae*	*Unclassified*	2.511	0.058	0.047	0.004
