# Supplementary material for: The equine gastrointestinal microbiome: impacts of weight-loss
Source: BMC Vet Res. 2020 Mar 4;16:78. doi: 10.1186/s12917-020-02295-6 (PMC7057583; doi:10.1186/s12917-020-02295-6)
Supplement: Supplementary file 13 — Additional File 13. Relative abundance of outset bacterial OTUs significantly different in abundance between low and mid weight-loss groups (n = 5/group). [file 12917_2020_2295_MOESM13_ESM.pdf]

Additional File 13. Relative abundance of outset bacterial OTUs significantly different in abundance between low and mid weight-loss groups (n = 5/group).

Phylum	Class	Order	Family	Genus	log2FoldChange	Adjusted P-value	Relative abundance	
							Mid	Low
Fibrobacteres	*Fibrobacteria*	*Fibrobacterales*	*Fibrobacteraceae*	*Fibrobacter*	8.824	0.000	2.229	0.008
Bacteroidetes	*Bacteroidia*	*Bacteroidales*	*Unclassified*	*Unclassified*	23.828	0.000	0.207	0.000
Bacteroidetes	*Bacteroidia*	*Bacteroidales*	*Bacteroidales_incertae_sedis*	*Phocaeicola*	20.864	0.000	0.012	0.000
Bacteroidetes	*Bacteroidia*	*Bacteroidales*	*Prevotellaceae*	*Unclassified*	25.166	0.000	0.373	0.000
Bacteroidetes	*Bacteroidia*	*Bacteroidales*	*Marinilabiliaceae*	*Alkalitalea*	25.081	0.000	0.264	0.000
Bacteroidetes	*Bacteroidia*	*Bacteroidales*	*Porphyromonadaceae*	*Unclassified*	23.452	0.000	0.084	0.000
Bacteroidetes	*Bacteroidia*	*Bacteroidales*	*Prevotellaceae*	*Unclassified*	23.663	0.000	0.132	0.000
Spirochaetes	*Spirochaetia*	*Spirochaetales*	*Spirochaetaceae*	*Treponema*	-22.438	0.000	0.000	0.085
Fibrobacteres	*Fibrobacteria*	*Fibrobacterales*	*Fibrobacteraceae*	*Fibrobacter*	8.255	0.001	0.070	0.000
Bacteroidetes	*Bacteroidia*	*Bacteroidales*	*Rikenellaceae*	*Unclassified*	3.523	0.001	0.260	0.028
Bacteroidetes	*Bacteroidia*	*Bacteroidales*	*Unclassified*	*Unclassified*	15.315	0.001	0.000	0.000
Fibrobacteres	*Fibrobacteria*	*Fibrobacterales*	*Fibrobacteraceae*	*Fibrobacter*	7.711	0.001	0.069	0.000
Bacteroidetes	*Bacteroidia*	*Bacteroidales*	*Unclassified*	*Unclassified*	6.675	0.002	0.045	0.000
Bacteroidetes	*Bacteroidia*	*Bacteroidales*	*Unclassified*	*Unclassified*	5.892	0.002	0.066	0.002
Bacteroidetes	*Bacteroidia*	*Bacteroidales*	*Prevotellaceae*	*Unclassified*	-3.049	0.003	0.009	0.103
Fibrobacteres	*Fibrobacteria*	*Fibrobacterales*	*Fibrobacteraceae*	*Fibrobacter*	10.972	0.009	1.163	0.001
Spirochaetes	*Spirochaetia*	*Spirochaetales*	*Spirochaetaceae*	*Treponema*	5.365	0.009	0.094	0.003
Bacteroidetes	*Bacteroidia*	*Bacteroidales*	*Unclassified*	*Unclassified*	3.026	0.010	0.075	0.011
Bacteroidetes	*Bacteroidia*	*Bacteroidales*	*Unclassified*	*Unclassified*	6.328	0.015	0.064	0.001

Bacteroidetes	*Bacteroidia*	*Bacteroidales*	*Unclassified*	*Unclassified*	4.648	0.021	0.133	0.007
Bacteroidetes	*Unclassified*	*Unclassified*	*Unclassified*	*Unclassified*	3.806	0.025	0.032	0.003
Firmicutes	*Clostridia*	*Clostridiales*	*Unclassified*	*Unclassified*	-4.598	0.032	0.000	0.020
Proteobacteria	*Alphaproteobacteri a*	*Unclassified*	*Unclassified*	*Unclassified*	-5.586	0.032	0.001	0.106
Fibrobacteres	*Fibrobacteria*	*Fibrobacterales*	*Fibrobacteraceae*	*Fibrobacter*	5.999	0.033	0.014	0.000
Bacteroidetes	*Unclassified*	*Unclassified*	*Unclassified*	*Unclassified*	6.240	0.034	0.051	0.001
Bacteroidetes	*Bacteroidia*	*Bacteroidales*	*Porphyromonadaceae*	*Unclassified*	5.192	0.035	0.977	0.030
Bacteroidetes	*Bacteroidia*	*Bacteroidales*	*Porphyromonadaceae*	*Unclassified*	7.737	0.038	0.054	0.000
Bacteroidetes	*Bacteroidia*	*Bacteroidales*	*Unclassified*	*Unclassified*	-5.150	0.038	0.001	0.048
Firmicutes	*Unclassified*	*Unclassified*	*Unclassified*	*Unclassified*	-3.586	0.038	0.007	0.105
Bacteroidetes	*Bacteroidia*	*Bacteroidales*	*Porphyromonadaceae*	*Paludibacter*	-4.899	0.038	0.011	0.388
Firmicutes	*Clostridia*	*Clostridiales*	*Unclassified*	*Unclassified*	3.461	0.038	0.024	0.003
Firmicutes	*Unclassified*	*Unclassified*	*Unclassified*	*Unclassified*	-5.450	0.046	0.000	0.024
Unclassified	*Unclassified*	*Unclassified*	*Unclassified*	*Unclassified*	-6.948	0.049	0.000	0.062
Unclassified	*Unclassified*	*Unclassified*	*Unclassified*	*Unclassified*	3.084	0.052	0.059	0.008
Firmicutes	*Clostridia*	*Clostridiales*	*Lachnospiraceae*	*Unclassified*	2.637	0.055	0.043	0.009
Bacteroidetes	*Bacteroidia*	*Bacteroidales*	*Unclassified*	*Unclassified*	-4.422	0.059	0.030	0.769
Bacteroidetes	*Bacteroidia*	*Bacteroidales*	*Prevotellaceae*	*Prevotella*	3.534	0.060	0.349	0.041
Firmicutes	*Clostridia*	*Clostridiales*	*Lachnospiraceae*	*Unclassified*	-2.685	0.060	0.025	0.189
Bacteroidetes	*Bacteroidia*	*Bacteroidales*	*Unclassified*	*Unclassified*	7.391	0.060	0.252	0.001
Firmicutes	*Clostridia*	*Clostridiales*	*Unclassified*	*Unclassified*	-3.184	0.060	0.004	0.042
Bacteroidetes	*Unclassified*	*Unclassified*	*Unclassified*	*Unclassified*	6.911	0.065	0.061	0.000
Firmicutes	*Clostridia*	*Clostridiales*	*Lachnospiraceae*	*Lachnospiracea _incertae_sedis*	2.024	0.079	0.025	0.008
Bacteroidetes	*Unclassified*	*Unclassified*	*Unclassified*	*Unclassified*	-7.511	0.087	0.000	0.095

Bacteroidetes	*Bacteroidia*	*Bacteroidales*	*Unclassified*	*Unclassified*	3.813	0.087	0.022	0.002
Bacteroidetes	*Bacteroidia*	*Bacteroidales*	*Rikenellaceae*	*Rikenella*	2.720	0.093	0.046	0.008
Bacteroidetes	*Unclassified*	*Unclassified*	*Unclassified*	*Unclassified*	2.003	0.093	0.025	0.007
Bacteroidetes	*Bacteroidia*	*Bacteroidales*	*Bacteroidales_incertae_sedis*	*Phocaeicola*	2.883	0.093	0.048	0.008
Bacteroidetes	*Bacteroidia*	*Bacteroidales*	*Unclassified*	*Unclassified*	1.752	0.093	0.073	0.026
