# Supplementary material for: The equine gastrointestinal microbiome: impacts of weight-loss
Source: BMC Vet Res. 2020 Mar 4;16:78. doi: 10.1186/s12917-020-02295-6 (PMC7057583; doi:10.1186/s12917-020-02295-6)
Supplement: Supplementary file 14 — Additional File 14. Relative abundance of outset bacterial OTUs significantly different in abundance between mid and high weight-loss groups (n = 5/group). [file 12917_2020_2295_MOESM14_ESM.pdf]

Additional File 14. Relative abundance of outset bacterial OTUs significantly different in abundance between mid and high weight-loss groups (n = 5/group).

Phylum	Class	Order	Family	Genus	log2FoldChange	Adjusted P-value	Relative abundance	
							Mid	High
Bacteroidetes	*Bacteroidia*	*Bacteroidales*	*Porphyromonadaceae*	*Unclassified*	21.576	0.000	0.099	0.000
Bacteroidetes	*Bacteroidia*	*Bacteroidales*	*Unclassified*	*Unclassified*	23.267	0.000	0.504	0.000
Bacteroidetes	*Bacteroidia*	*Bacteroidales*	*Porphyromonadaceae*	*Unclassified*	10.890	0.000	0.977	0.000
Bacteroidetes	*Bacteroidia*	*Bacteroidales*	*Porphyromonadaceae*	*Unclassified*	9.202	0.000	0.460	0.000
Bacteroidetes	*Bacteroidia*	*Bacteroidales*	*Unclassified*	*Unclassified*	-23.064	0.000	0.000	0.049
Bacteroidetes	*Unclassified*	*Unclassified*	*Unclassified*	*Unclassified*	-23.526	0.000	0.000	0.070
Bacteroidetes	*Bacteroidia*	*Bacteroidales*	*Porphyromonadaceae*	*Unclassified*	22.386	0.000	0.270	0.000
Bacteroidetes	*Bacteroidia*	*Bacteroidales*	*Unclassified*	*Unclassified*	-23.187	0.000	0.000	0.062
Spirochaetes	*Spirochaetia*	*Spirochaetales*	*Spirochaetaceae*	*Treponema*	-22.846	0.000	0.000	0.045
Firmicutes	*Clostridia*	*Clostridiales*	*Lachnospiraceae*	*Unclassified*	-8.121	0.000	0.001	0.172
Bacteroidetes	*Unclassified*	*Unclassified*	*Unclassified*	*Unclassified*	-6.884	0.006	0.001	0.068
Firmicutes	*Clostridia*	*Clostridiales*	*Ruminococcaceae*	*Unclassified*	-4.622	0.009	0.107	1.364
Proteobacteria	*Alphaproteobacteria*	*Unclassified*	*Unclassified*	*Unclassified*	-6.280	0.009	0.001	0.134
Bacteroidetes	*Bacteroidia*	*Bacteroidales*	*Unclassified*	*Unclassified*	-7.864	0.016	0.001	0.069
Firmicutes	*Clostridia*	*Clostridiales*	*Ruminococcaceae*	*Flavonifractor*	-3.498	0.022	0.007	0.037
Bacteroidetes	*Bacteroidia*	*Bacteroidales*	*Porphyromonadaceae*	*Barnesiella*	-4.058	0.042	0.356	2.519
Bacteroidetes	*Cytophagia*	*Cytophagales*	*Unclassified*	*Unclassified*	-4.964	0.046	0.021	0.379
Firmicutes	*Unclassified*	*Unclassified*	*Unclassified*	*Unclassified*	3.848	0.053	0.064	0.002
Firmicutes	*Clostridia*	*Clostridiales*	*Lachnospiraceae*	*Unclassified*	-3.969	0.058	0.007	0.066

Spirochaetes	*Spirochaetia*	*Spirochaetales*	*Spirochaetaceae*	*Treponema*	-7.295	0.066	0.000	0.056
Bacteroidetes	*Bacteroidia*	*Bacteroidales*	*Unclassified*	*Unclassified*	2.851	0.066	0.078	0.005
Firmicutes	*Clostridia*	*Clostridiales*	*Ruminococcaceae*	*Oscillibacter*	-3.696	0.066	0.007	0.041
Spirochaetes	*Spirochaetia*	*Spirochaetales*	*Spirochaetaceae*	*Treponema*	6.298	0.081	0.064	0.000
Bacteroidetes	*Unclassified*	*Unclassified*	*Unclassified*	*Unclassified*	6.918	0.091	0.061	0.000
Bacteroidetes	*Bacteroidia*	*Bacteroidales*	*Unclassified*	*Unclassified*	2.908	0.094	0.036	0.003
Bacteroidetes	*Bacteroidia*	*Bacteroidales*	*Unclassified*	*Unclassified*	-5.870	0.100	0.003	0.102
Firmicutes	*Negativicutes*	*Selenomonadales*	*Acidaminococcaceae*	*Phascolarctobacterium*	-4.560	0.100	0.003	0.043
Bacteroidetes	*Unclassified*	*Unclassified*	*Unclassified*	*Unclassified*	-6.900	0.100	0.001	0.039
