# Supplementary material for: The equine gastrointestinal microbiome: impacts of weight-loss
Source: BMC Vet Res. 2020 Mar 4;16:78. doi: 10.1186/s12917-020-02295-6 (PMC7057583; doi:10.1186/s12917-020-02295-6)

Additional File 15. Principal Co-ordinate analysis plot of the post-diet bacterial community from the three weight-loss groupings using Bray-Curtis similarity index.

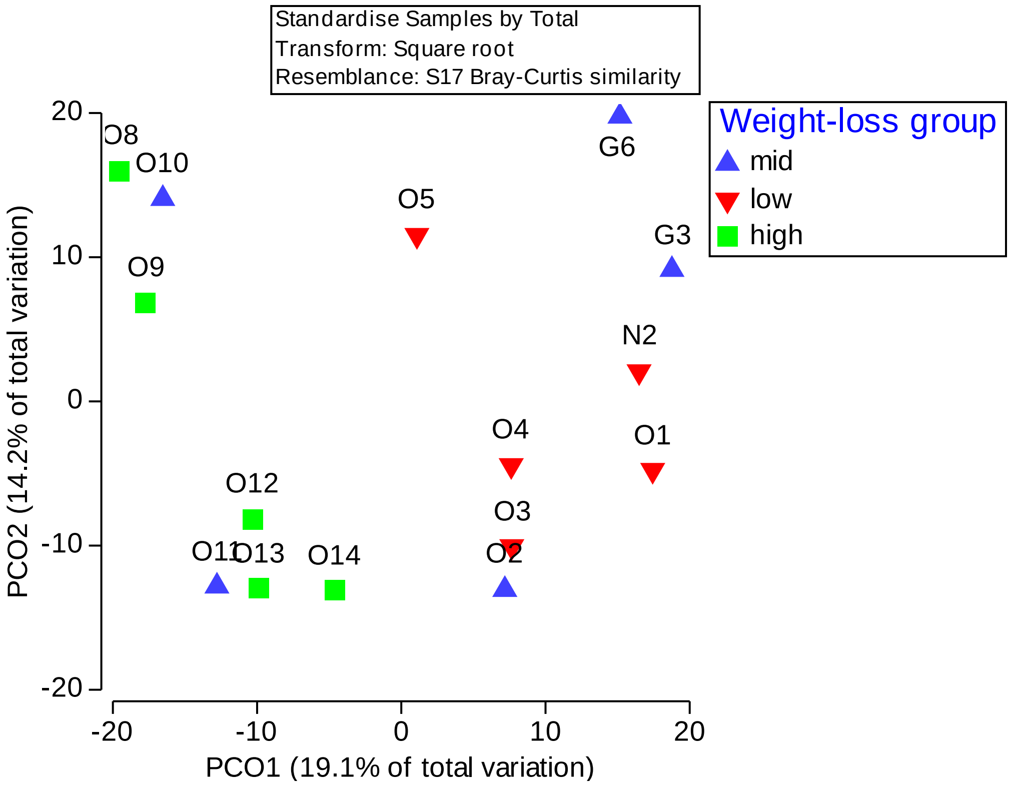

Supplement: Supplementary file 15 — Additional File 15. Principal Co-ordinate analysis plot of the post-diet bacterial community from the three weight-loss groupings using Bray-Curtis similarity index. [file 12917_2020_2295_MOESM15_ESM.pdf]
